# Clinicians’ use of the structured professional judgement approach for adult secure psychiatric service admission assessments: A systematic review

**DOI:** 10.1371/journal.pone.0308598

**Published:** 2024-09-26

**Authors:** Jana Bowden, Caroline Logan, Louise Robinson, Jon Carey, James McDonald, Ruth McDonald, Jennifer Shaw, Jane Senior, Sarah Leonard

**Affiliations:** 1 The University of Manchester, Manchester, United Kingdom; 2 Greater Manchester Mental Health NHS Foundation Trust, United Kingdom; 3 Lancashire and South Cumbria NHS Foundation Trust, United Kingdom; 4 Northumbria University, Newcastle upon Tyne, United Kingdom; 5 Newcastle University, Newcastle upon Tyne, United Kingdom; University of Pennsylvania, UNITED STATES OF AMERICA

## Abstract

The structured professional judgement (SPJ) approach was initially developed to support risk assessment and management decisions. The approach is now being adapted and applied to admission assessments for adult secure services. This systematic review aims to summarise the evidence for the effectiveness and acceptability of the SPJ approach in admission assessments of this kind. A comprehensive electronic search strategy was used to identify studies indexed in PubMed, PsycInfo, Medline and Cochrane Library (January 2007 –January 2024). Two search strategies included terms (and synonyms) for psychiatric patients (quantitative) or clinicians and clinicians’ experiences (qualitative), structured professional judgement, admission, and secure services. Twelve quantitative articles (published 2007–2020) were identified. SPJ-informed guidance included were the DUNDRUM-1, DUNDRUM-2, DUNDRUM-3, DUNDRUM-4, and the HCR-20. While findings were variable, the overall pattern indicated that ratings suggestive of more problems were associated with increased likelihood of admission or movement to higher security levels. There is emerging evidence for the use of SPJ guidance to support admission decision-making. Specifically, it should be used as an adjunct to existing decision-making processes rather than as a replacement for those processes. Further research, both quantitative and qualitative, across a wider range of settings and populations is recommended.

## Introduction

Secure psychiatric services (SPS) provide intensive hospital care, treatment and rehabilitation for those who are mentally unwell and display high risk behaviours. Due to differing legal frameworks, policies and administrative procedures, the composition of these services varies internationally, leading to heterogeneity in the service users that are admitted to SPS [1, 2]. Many countries have some form of specialised secure or forensic facilities; however, in some instances, general psychiatric hospitals are the primary treatment facilities for all service users regardless of concerns about risk [3]. While differences in how secure services or their equivalents operate internationally may be expected, there is also regional variability. For example, in Australia, New Zealand, Canada and the USA, there is no national structure, and psychiatric services are variable across regions or states [3]. This is, to some extent, like Germany, Belgium and the Netherlands, where service provision is district or county based, while in Austria, service provision is centralised [3].

### The context in England and Wales

In England and Wales, SPS treat people with severe mental health problems who are deemed to be a serious risk to others. This includes those who are subject to criminal proceedings and those who cannot be safely cared for in general, non-secure mental health settings. SPS are commissioned at three security levels: low, medium and high, with the level of security determined by a service user’s assessed level of risk [4]. Referrals to SPS originate from community settings (including police custody], secondary mental health services, other SPS or the criminal justice system (subject to Part 3 of the Mental Health Act. *revised 2007*) [5]. Upon receipt of a referral, service users receive an ‘access assessment’ to determine if the criteria for admission to secure care are met and to consider any alternatives to admission to such a setting. Where admission is indicated, the appropriate level of security should be identified according to the principle of ‘least restrictive practice’ (as outlined in the Mental Health Act. *revised 2007*) [5].

Establishing who is eligible for admission to SPS is complex. Admission criteria are inconsistently applied and are dependent on a range of regional (e.g., variations in local population characteristics and individual SPS provision) [6] and individual factors (e.g., the nature of an individual’s disorder, the notoriety of their offence, lack of alternative arrangements) [7]. This disparity means that generalisation from studies of single SPS is not reliable. There are few national studies of SPS, and there is just one study from 2004 that has focused on factors associated with admission and acceptance for care [8]. This study captured data from 418 admission assessments across 34 medium SPS in England and Wales and examined the ways in which such services prioritised admissions [8]. Of those assessed as requiring medium security, 82% were accepted or placed on the waiting list [8]. In total, 77% of those accepted or placed on the waiting list for medium security were those assessed as requiring medium security while 23% were initially assessed as needing a lower security level [8]. This indicates that service users may be cared for in services with higher security restrictions than required, or possibly that security level requirements can change after an initial assessment [8]. The authors concluded that: (i) overall, more people require admission to medium SPS than can be accommodated; and (ii) the insufficient range of SPS provision available leads to the inappropriate use of medium SPS beds, where those who are assessed as needing lower levels of security are admitted over those who are considered to require long-term medium security [8]. This pattern has been observed across levels of security since the early 1990s [8–14]. There is a lack of research conducted more recently and the length of time since these studies were conducted limits current applicability and relevance, especially given continual developments in secure service provision.

Little is known about clinical decision-making in the context of SPS admission assessments. However, early qualitative research with clinicians working within SPS suggested that a range of contextual factors and individual clinician values and assumptions guide and influence who is accepted for admission to SPS [15]. Clinicians described decisions to admit as ‘discretionary professional judgements’, which are in part influenced by extraneous factors, such as relationships with external colleagues, policy context, an excess of demand over availability of beds, and the need to ensure turnover [15]. Whilst important for service management, these factors may not be directly relevant to the immediate treatment needs of an individual service user and might contribute to the inequity of access to SPS. This raises the question whether approaches to clinical decision-making when assessing for admission should account for extraneous factors or should be standardised with reduced influence of subjective judgement.

NHS England and NHS Improvement now delegate specified commissioning responsibilities for adult low and medium SPS to regional Provider Collaboratives (PCs) [16]. PCs involve partnerships between two or more trusts with one aim being reduction of inequality in outcomes and access to services [16]. Embedded within the PC model is the Access Assessment Service (AAS). This a single point of access referral pathway which streamlines the referral and assessment process, with the aim of reducing multiple assessments and delays. The standards for carrying out access assessments are outlined in the service specifications for low and medium SPSs [17, 18]. These standards describe how the AASs are responsible for delivering against specific timescales and that access assessments should be conducted within a structured framework which includes validated assessment guidelines [17, 18].

### Assessment approaches

Assessment approaches in clinical practice, specifically for the assessment and management of violence risk in those with mental health problems, have developed extensively in the last 30 years. In England and Wales, the SPJ approach is proposed as a practical and standardised framework for clinical risk assessment and management [19, 20]. This approach focuses on harm prevention through an understanding of individual risk and what is required to address the risk and protective factors most relevant to mitigating adverse outcomes [21]. The SPJ approach is embodied in evidence-based guidelines or frameworks (they may also be referred to as ‘tools’ or ‘instruments’) that promote systematisation and consistency but are centred around discretionary rather than formulaic decision-making and are flexible enough to account for case-specific influences and the contexts in which assessments are conducted [22, 23]. The SPJ approach was developed because of the limitations of alternative approaches, specifically unstructured clinical judgement and non-discretionary formulaic approaches [23]. Unstructured clinical judgement involves clinician discretion and instinct in the absence of formal guidance [23]. As the unstructured approach lacked reliability and predictive accuracy, the non-discretionary approach was developed to increase reliability and validity, with set rules on what to consider when evaluating risk [23]. Using this approach, algorithmic instruments known as actuarial risk assessment instruments (ARAIs) are used to score the likelihood of a specific risk outcome and categorise individuals according to risk [23]. While reliability and predictive accuracy improved as a result of this development, this approach lacked flexibility and could not account for individual characteristics and context, such as different settings and change over time [23]. In light of these limitations, the SPJ approach was evolved to combine discretion with evidence-based guidelines [23]. The approach is particularly suited to the risk assessment and management task because the structure offered ‘helps to mitigate the biases and errors that plague all decisions made under conditions of uncertainty’ [24, p. 320]. There is an extensive development process for the development of a set of SPJ guidance, with recent examples including support for key assessment tasks such as risk formulation and scenario-planning, as well as comprehensive risk management planning.

While SPJ has a specific meaning in relation to risk assessment and management, the core principles of the SPJ approach are being reflected in other areas of practice involving complex decision-making, leading to subtle variations in how the term ‘SPJ’ is used. For example, the combination of explicit evidence to guide assessment, structure underpinning the decision-making process, and systematisation (which helps to increase transparency, reliability, accountability, and consistency), alongside maintaining the importance of discretionary clinical judgement, have inspired developments in admission decision-making [23, 25]. These developments have followed a similar trajectory to that for risk assessment and management. There have been attempts to move away from the established practice of using discretionary unstructured clinical judgement in admission decision-making to develop a standardised and reliable approach to the SPS admission assessment process. Thus, formulaic instruments focusing on the security and placement needs of those referred to SPS and based on clear and objective criteria for each security level [7, 26–28] were followed by guidance based on the SPJ approach [29, 30]. One such example is the Dangerousness, Understanding, Recovery and Urgency Manual (DUNDRUM), consisting of four sets of guidelines designed to support decisions related to admission and step-up/step-down security level movement [25]. The DUNDRUM toolkit has broadly adopted the principles of the SPJ approach [25]; while items in the DUNDRUM guidelines can be scored by clinicians–scoring is no longer encouraged in SPJ risk guidance–this is to support clinical judgement rather than produce a decision using an algorithm or ‘cut-off’ score [25].

There is an extensive international literature that provides evidence for how the SPJ approach, embodied in guidance, can be used effectively for risk assessment and management [31–33]. This body of research has to date focused on understanding risk in the individual case and using that understanding to guide interventions and therefore limit if not prevent further harmful behaviour. The research has improved general understanding of risk and protective factors alongside interventions that impact on harm prevention. This is to be expected as this is the context in which the SPJ approach was developed. However, SPJ risk guidance is being used in admission decision-making, and it remains to be seen how applicable this is in this context. In this review, the term SPJ approach is not limited to the SPJ approach used in the risk assessment and management literature. Instead, its use in this paper intends to convey a broader meaning where core SPJ principles, specifically a combination of discretion and systemisation, have been applied to admission decision-making to ensure consistent, accountable and evidence-based practice.

This paper involves a systematic search and narrative synthesis of international research concerned with the applications of the SPJ approach in SPS admission assessments and decisions concerning changes in level of security need. It is intended that findings from this review will provide a knowledge base that will help inform the future implementation of the SPJ approach in admission assessment practice for SPS, in England and Wales and also internationally.

## Methodology

We collated all relevant studies concerned with the effectiveness and acceptability of guidance based on the SPJ approach for the purpose of admission assessments to adult SPS. The protocol for this review was registered on the *International Prospective Register of Systematic Reviews* (CRD42022351425) [34].

### Study inclusion/exclusion criteria

#### Study design

All study designs were eligible for inclusion in this review, including quantitative, qualitative and mixed-methods studies. Before the completion of the searches, it was unclear whether a range of designs had been used to investigate this research area. Separate search strategies for quantitative and qualitative data were designed to identify all potentially relevant research, regardless of research design. This review also included a grey literature search.

#### Population

Quantitative search: Service users admitted to secure psychiatric services.

Qualitative search: SPS clinicians, such as nurses, psychiatrists and psychologists, involved in admission assessments using SPJ guidance. The only participant criteria was a clinical role in the SPS admission process. Therefore, clinicians working in non-secure inpatient services, community services or children’s services were excluded.

#### Service type

The focus of this review was specifically adult SPS. This included low, medium or high secure services within the United Kingdom, alongside equivalent services outside of the United Kingdom. Non-secure adult inpatient services, community services and secure services for children and young people were excluded.

#### Assessment approach

Included in the review were all types of SPJ guidance specifically utilised in the context of assessment for admission to secure psychiatric services (including movements between levels of security). Admission assessments that were not based on the SPJ approach (for example, they were based instead on the use of ‘checklists’, or actuarial approach) or SPJ guidance used in a context other than for admission to secure psychiatric services were excluded.

#### Assessment period

The assessment period included was either upon admission or when moving through levels of security. The admission of service users moving through levels of security following a SPJ-based assessment effectively represents a “new” admission to secure psychiatric services, i.e., at a different security level to the previous admission. As a result, studies were included if the focus was on assessments based on the SPJ approach upon admission to secure care or to determine whether needs had changed regarding security level requirements. Excluded were discharge assessments or routine non-admission assessments (e.g., for ongoing patient care team reviews).

### Literature search

#### Electronic digital databases

The systematic search was conducted across the databases PubMed, PsycInfo, Medline, and Cochrane Library. The search focused on research published from January 2007 to August 2022, when the initial search was conducted. As the application of the SPJ approach to admission assessments has developed more recently, with the DUNDRUM Manual published in 2013, a 15-year search period was judged to be sufficient. The two discrete search strategies were initially piloted in PubMed and were adapted slightly for other databases to match subject headings specific to each platform. The quantitative search strategy included terms related to a population of psychiatric patients, structured professional judgement, admission, and secure services (see Table 1 of search terms). The qualitative search strategy included terms related to a population of clinicians, structured professional judgement, clinicians’ experiences, admission, and secure psychiatric services (see Table 2 for search terms). Searches were limited to English language studies but not a specific country. Searches were repeated in January 2024 to ensure the most recent research was included.

**Table 1:** Quantitative search strategy.

Psychiatric patients		Structured professional judgement		Admission		Secure service
**OR**		**OR**		**OR**		**OR**
Inpatients		Assess*		Psychiatric hospital admission		Security level
**OR**		**OR**		**OR**		**OR**
Hospitalised^2^/ Hospitalized^1^/ Hospitali*ed^3^ patients		Risk* (OR Risk assessment OR risk management^1^)		Access		Low secure
	**AND**	**OR**	**AND**	**OR**	**AND**	**OR**
		Decision-making		Gatekeep*		Medium secure
		**OR**		**OR**		**OR**
		Screening tools		Refer*		High secure
		**OR**				**OR**
		Clinical judgement (Not diagnosis)^1^				Psychiatric service
						**OR**
						Secure care
						**OR**
						Mental Health Services^1^

Pubmed quantitative search strategy with slight modifications shown for each database.

*Note*. All searches were limited to research published from 2007 onwards.

^1^ Psycinfo and Medline specific subject headings

^2^ Pubmed strategy

^3^ Cochrane strategy

**Table 2:** Qualitative search strategy.

Clinicians		Structured professional judgement		Opinion		Admission		Secure service
**OR**		**OR**		**OR**		**OR**		**OR**
Professionals		Assess*		Attitude		Psychiatric hospital admission		Security level
**OR**		**OR**		**OR**		**OR**		**OR**
Psychiatrists	**AND**	Risk* (OR risk assessment OR risk management^1^)	**AND**	Judge	**AND**	Access	**AND**	Low secure
**OR**		**OR**		**OR**		**OR**		**OR**
Psychologists		Decision-making		View		Gatekeep*		Medium secure
**OR**		**OR**		**OR**		**OR**		**OR**
Nurs*		Screening tools		Perspective		Refer*		High secure
		**OR**		**OR**				**OR**
		Clinical judgement (Not diagnosis)^1^		Experience				Psychiatric service
								**OR**
								Secure care
								**OR**
								Mental Health Services^1^

Pubmed qualitative search strategy with slight modifications shown for each database.

*Note*. All searches were limited to research published from 2007 onwards.

^1^ Psycinfo and Medline specific subject headings

### Grey literature

Grey literature was identified using Google and Google Scholar (first 100 outputs), alongside reading articles the research team were already aware of and searching reference lists of included articles. These alternative search techniques are recommended to ensure inclusion of all relevant articles in the review [35]. This is particularly important due to the possibility of limited evidence in this area.

### Study selection

**Fig 1:**
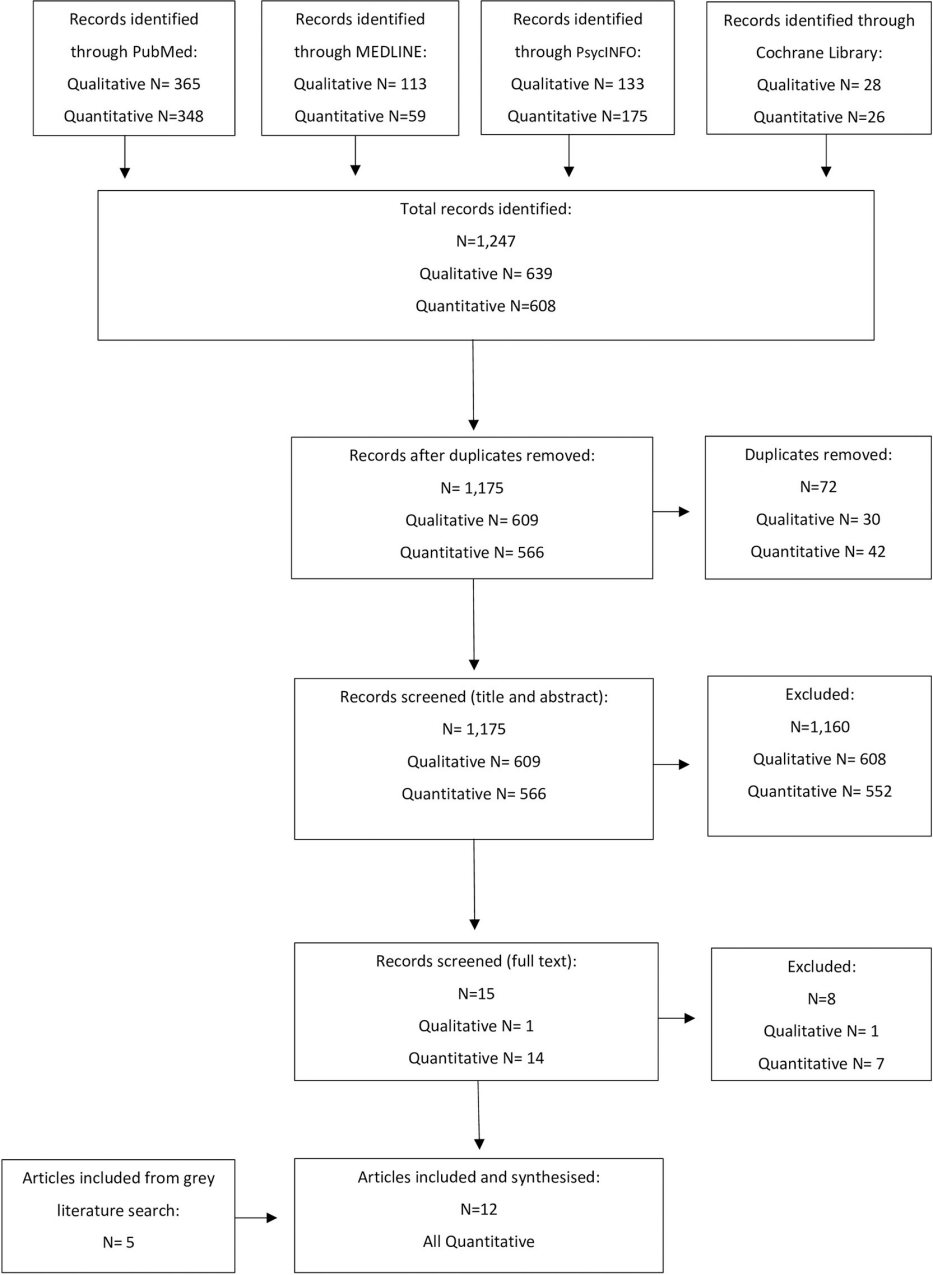
PRISMA flow diagram, adapted based on PRISMA guidance [36].

Articles returned from each database search were exported to Endnote (2013) to allow cross-referencing for duplicates. A list of titles was then extracted into Microsoft Excel. Titles and abstracts were screened independently by two members of the research team based on the inclusion and exclusion criteria. Reviewers first independently screened 5% of the abstracts and met to assess screening consistency before independently screening the remaining abstracts. Once screening was complete, reviewers cross-referenced articles identified as fitting the inclusion/exclusion criteria. In instances of disagreement around inclusion or exclusion, a full-text review was conducted. If an agreement was not reached following full-text review, a senior member of the research team decided whether to include the article. This title and abstract screening was followed by full-text review of the included articles to confirm eligibility for data extraction. This was completed by one member of the research team with support from another researcher where input was required. The screening and selection process is further illustrated in the PRISMA flow diagram [36] in Fig 1. As no qualitative articles were included following the full-text screen, the planned methodology for extracting and synthesising qualitative research was not utilised. Please see the published PROSPERO protocol (CRD42022351425) for the anticipated methodology [34].

### Data extraction

One member of the research team extracted data from included articles. Extracted data provides insight into the use of the SPJ approach for admission assessments. This included the accuracy of the SPJ approach to inform admission decision-making. Endnote and Microsoft Excel were utilised to aid data extraction. The extracted data included: author, country, year, study title, research aim, study design, methodology, clinical setting, level of security, client group, sample size, sample characteristics, SPJ guidance utilised, outcome measure(s) and findings, alongside the accuracy of the SPJ approach to guide admission.

### Quality assessment

The Mixed Methods Appraisal Tool (MMAT) was selected to conduct quality appraisal due to utility across a range of study designs including qualitative, quantitative (descriptive, randomised, and non-randomised) and mixed-methods studies [37]. Due to the inclusion criteria not limited by study design, this tool was judged to be appropriate for the purposes of the review. While only quantitative studies were identified from the search, this tool remained suitable by accounting for a range of quantitative designs, allowing all studies to be appraised using comparable criteria. The MMAT criteria includes the clarity of the research question, the suitability of the selected design to address the research question, participant selection, analysis, and interpretation [37]. The clarity of the research question was considered for all study designs, whilst the remaining criteria to assess study quality were specific based on research design. The tool does not employ a numerical scoring system, rather a more detailed judgement regarding quality is reached based on whether the answer was yes/no/not clear to the criteria. A judgement identifying limitations and areas for future research to improve was considered more helpful than a numerical score for this review, particularly given the possibility of limited research in the area. Two reviewers conducted the quality assessment, with a third resolving any disagreements. The quality was variable across studies, although certain limitations were more prevalent such as a lack of accounting for confounding variables, lack of clarity regarding outcome measures and lack of information regarding the selected sample. All studies were included, regardless of perceived quality, as recommended when using the MMAT [37] and to present the current evidence base completely. However, the limitations are highlighted and discussed. The complete quality appraisal is presented as supporting information [S1 and S2 Tables].

### Analysis

This paper was a systematic review that involved no analysis of data.

### Data synthesis

The minimum number of studies required for data synthesis in this review was one. Findings were first grouped by SPJ guidance and then further grouped by outcome. Outcomes from the included studies are summarised to provide an overview of the current use of SPJ-informed guidance for secure service admission, which is focused on the accuracy of the SPJ approach to inform admission decision-making. The synthesis was guided by the Synthesis Without Meta-analysis (SWiM) [38]. The SWiM guidance “is a nine-item checklist to promote transparent reporting for reviews of interventions that use alternative synthesis methods of quantitative studies” [38, p. 1].

## Results

### Description of full-text articles retrieved

The systematic search identified 1,247 articles, published between January 2007 and August 2022. Following removal of duplicates and screening, seven quantitative articles were identified as relevant to the review [39–45]. Five further quantitative articles were identified as relevant for the review through the grey literature search [46–50] resulting in a total of 12 articles included for quality appraisal and synthesis. Fig 1 shows the search process and how many articles remained following each stage [36]. No further articles met inclusion criteria when searches were repeated prior to completion of the review in January 2024. Extracted information for each study is presented in Table 3, grouped by study design. SWiM guidance recommends grouping studies to allow comparison and meaningful synthesis of findings across articles with a variety of research designs [38]. The extracted information was synthesised using narrative synthesis with findings grouped by SPJ guidance [51].

**Table 3:** Information extracted from included articles, grouped by study design.

Group	Study background: Author(s) Year Study title	Country	Aims	Study design and Methodology	Referral source, Admission setting and Level of security	Client group, Sample size and Sample characteristics	SPJ guidance and Outcome measure	Findings	Accuracy of the SPJ approach to guide admission
Retrospective	G. Flynn; C. O’Neill; C. McInerney; H.G. Kennedy 2011 The DUNDRUM-1 structured professional judgment for triage to appropriate levels of therapeutic security: retrospective-cohort validation study	Republic of Ireland	To examine the validity of the DUNDRUM-1 for indicating admission to psychiatric hospital and distinguishing between required security level. To examine the inter-rater reliability of the DUNDRUM-1	Design Retrospective cohort Methodology Two authors used the DUNDRUM-1 to rate a sample of those diverted from prison to psychiatric hospital and a sample identified as requiring further assessment. Both authors were blind to outcome when rating both samples. Unstructured pre-admission assessments and court reports were used by the clinicians to rate the DUNDRUM-1. Each DUNDRUM-1 item was rated on a 5-point scale from 0 (no security need) to 4 (requiring special/high security). The ratings on the DUNDRUM-1 were then compared to the patient outcomes from a prison in-reach and court diversion scheme regarding whether they were admitted and level of security.	Referral source: One remand prison (Clover Hill) in Dublin, Ireland Admission setting: Local hospitals (open), Low secure units, Central Mental Hospital (medium and high)	N = 316 2-year sample: All new committals diverted from the prison to psychiatric hospital (January 2008- December 2009). N = 100 3-month sample: All new committals to the prison identified for full psychiatric assessment (April—June 2009). N = 246 (N = 30 overlapped between samples). Overall sample characteristics: Male, further characteristics not reported.	DUNDRUM-1 (11-items) The predictive validity of the DUNDRUM-1: Comparison of patient placement and total scores on the DUNDRUM-1. Receiver operating characteristics (ROC) and whether the Area Under the Curve (AUC) indicated that the DUNDRUM-1 distinguished between those admitted by security level. Inter-rater reliability 2-year sample diverted from prison outcomes: Admitted to an open hospital ward. Admitted to local PICU (locked low secure unit). Admitted to Central Mental Hospital (medium/high). 3-month sample identified for full psychiatric assessment outcomes: Discharged to the prison GP. Followed up by the prison psychiatric in-reach team or community mental health team. Admitted to any of the available hospitals.	2-year sample, diverted from prison to psychiatric hospital: Comparison of DUNDRUM-1 total score across the three placement outcomes indicated there was a significant effect of DUNDRUM-1 score on admission security level (ANOVA F = 75.2, df = 2, p<0.001)^a^. Those admitted to medium and high secure levels had a higher DUNDRUM-1 total score (mean = 22.87, SD = 4.56) than those admitted to PICU (mean = 15.81, SD = 4.39) or open wards (mean = 10.74, SD = 3.26). Differences in total DUNDRUM-1 scores were significant between each individual group (no Bonferroni statistics provided). 3-month sample, identified for full psychiatric assessment: Comparison of DUNDRUM-1 total score across the three assessment outcomes indicated there was a significant effect of DUNDRUM-1 score on patient outcome (ANOVA F = 360.1, df = 2, p<0.001). Those admitted to psychiatric hospital had a higher DUNDRUM-1 score (mean = 15.77, SD = 5.33) than those that were not admitted and received psychiatric follow-up (mean = 4.14, SD = 4.57) or were discharged to the prison GP (mean = 0.21, SD = 0.79). Differences in total DUNDRUM-1 scores were significant between each group (no Bonferroni statistics provided). Inter-rater reliability 18 cases were rated blindly and independently by two clinicians: The Kappa statistic for 7/11 items was higher than 0.85 for each item (p < .001). There was a significant correlation between DUNDRUM-1 scores of both clinicians for all 11 items (correlation greater than r_s_ = 0.75, p < .001) Total score (for 11 items) of both clinicians was significantly correlated (r_s_ = 0.96, p < .001).	DUNDRUM-1 predictive validity 2-year sample, diverted from prison to psychiatric hospital: ROC: Open wards v. psychiatric intensive care units: (AUC) = 0.81 (95% Confidence interval (CI) 0.68 to 0.93) Psychiatric intensive care units v. medium/high security (AUC) = 0.87 (95% CI 0.78 to 0.95) 3-month sample, identified for full psychiatric assessment: ROC: Discharge to GP v. follow-up/ admission: (AUC) = 0.89 (95% CI 0.84 to 0.94) Admitted v. not admitted: AUC = 0.98 (95% CI 0.97 to 0.98)
	D. Lawrence; T. L. Davies; R. Bagshaw; P. Hewlett; P. Taylor; A. Watt 2018 External validity and anchoring heuristics: application of DUNDRUM-1 to secure service gatekeeping in South Wales	Wales	To examine the validity of the DUNDRUM-1 for indicating admission to psychiatric hospital and distinguishing between required security level.	Design: Retrospective cohort Methodology: One of two authors used the DUNDRUM-1 to rate first-time referrals to secure services. Both authors were blind to outcome when rating the DUNDRUM-1. Ratings were based on patient characteristics extracted from a database of written gatekeeping assessment reports from January 2010- June 2013. The DUNDRUM-1 ratings were compared to patient outcomes regarding whether a referral was admitted and the required security level.	Referral source: Not specified Admission setting: Psychiatric services (Open, low, and medium secure)	N = 121 All first-time secure service referrals in South Wales (January 2010- June 2013) with a psychiatric report available and a record of their destination. Overall sample characteristics: Further characteristics not reported.	DUNDRUM-1 (11-items) The external and predictive validity of the DUNDRUM-1: Comparison of patient placement and scores on the DUNDRUM-1. ROC and whether the AUC indicated that the DUNDRUM-1 distinguished between those admitted to low/medium secure and those not admitted. DUNDRUM-1 outcomes were measured on a 5-point scale from (0) very low severity–(4) high severity. Security levels were recommended based on these ratings: (0) Community (1) Open (2) Acute low secure/PICU (3) Medium security (4) High security.	Comparison of the DUNDRUM-1 total score across the three placement outcomes (open, low, and medium) showed an overall significant difference between scores across security levels (Kruskal Wallis; H = 0.74, df = 2, p<0.000). Post-hoc alpha adjusted Mann-Whitney analyses indicated significantly lower scores for those admitted to: Open (mean = 20) v. low secure (mean = 23.86; p<0.050) Low v. medium secure (mean = 29.64; p<0.005)^b^ Open v. medium secure (p < .005). ^d^	DUNDRUM-1 predictive validity ROC: Admitted to secure services (low/medium) v. not admitted: AUC 0.76 (95% CI 0.65–0.85)^d^
	I. Jeandarme; P. Habets; H. Kennedy 2019 Structured versus unstructured judgment: DUNDRUM-1 compared to court decisions	Belgium	To examine the validity of the DUNDRUM-1 for distinguishing between required security level.	Design: Retrospective cohort Methodology: The second author scored the DUNDRUM-1 for all files (psychosocial reports while detained, psychiatric evaluation reports and treatment reports). Two random samples either referred to medium secure or various security levels were rated. The reports of those referred to medium secure were blindly scored for current placement but not blind to placement on admission. The reports for those referred to various security levels were scored blindly for court decision. DUNDRUM-1 ratings were compared to court decisions regarding patient placement.	Referral source: Court diversion Admission setting: Psychiatric services (medium secure specifically alongside other security levels)	N = 150 Sample 1, Court referrals for admission to a medium secure unit (OPZC Rekem), randomly selected: N = 50 Sample 2, Referrals for admission to various security levels, randomly selected: N = 100 It was not clear how sample size was selected. N = 5 excluded due to missing data Overall sample characteristics: Male, Mean age = 41.5 years (SD = 11.26, range 21–71 years), 86.6% Belgian nationality	DUNDRUM-1 (9-items) The external and predictive validity of the DUNDRUM-1: Comparison of court determined patient placement and mean scores on the DUNDRUM-1 (9-item version) ROC and whether the AUC indicated that the DUNDRUM-1 distinguished between different security levels. Level of agreement and correlation between court decisions and DUNDRUM-1 recommendations. Both Court decisions and DUNDRUM-1 scored on the DUNDRUM-1 security level scale from 0–4: Ambulatory Open Low Moderate (including MSU) High	Comparison of DUNDRUM-1 mean scores for different court decisions showed a significant difference between scores across groups overall (Kruskal Wallis, p < 0.05). Post-hoc analyses indicated significantly higher mean DUNDRUM-1 scores for those admitted to: High (M = 2.53, SD = 0.59) v. open (M = 1.60, SD = 0.37; p<0.01) High v. medium (M = 2.19, SD = 0.57; p<0.01). Level of agreement There was a weak significant correlation between DUNDRUM-1 and court recommendations (r = 0.25, p<0.05). There was agreement between DUNDRUM-1 and court decisions in 40% of cases. Court decisions indicated a higher security level than the DUNDRUM-1 in 45.5% of cases and lower security in 14.5% of cases. Kappa statistics indicated agreement was generally poor (k = 0.06, p = 0.15). Medium secure decisions represented the most agreement between DUNDRUM-1 scores (64.9%) and court decisions (52.7%) while there was no agreement for ambulatory/ open/low secure settings.	DUNDRUM-1 predictive validity ROC: Predictive of admission to a high security unit (AUC = 0.70; p < 0.05)^b^. Not predictive of medium secure admission Not predictive of court decision to admit to either medium or high secure.
	H. K. Williams; M. Senanayke; C. C. Ross; R. Bates; M. Davoren 2020 Security needs among patients referred for high secure care in Broadmoor Hospital England	England	To examine security needs of patients referred to Broadmoor high secure hospital using DUNDRUM-1 and DUNDRUM-2.	Design: Retrospective cohort Methodology Two authors blindly scored the DUNDRUM-1 and DUNDRUM-2 for all Broadmoor referrals. DUNDRUM scores were compared to outcomes of the standard admission process (assessments by a consultant forensic psychiatrist or specialist registrar, forensic mental health social worker and admissions panel). All assessors were blind to other scores or outcomes. DUNDRUM-1 ratings were compared to the recommendations made at each stage of the admissions process.	Referral source: Medium secure services (41.7%), Prison (58.3%) Admission setting: Broadmoor high secure hospital	N = 204 All referrals for admission to Broadmoor high secure hospital over a 2-year period (2015–2017). Overall sample characteristics: Male, Over 18 years old, Mean age (referred from medium secure) = 34.22 years (SD = 9.5), Mean age (referred from prison) = 33.98 years (SD = 11.5) 54.1% schizophrenia diagnosis, 15.1% personality disorder diagnosis, 26.8% unclear diagnosis. Diagnostic uncertainty more prevalent for prison (41.2%) v medium secure service referrals (7.1%).	DUNDRUM-1 (9-items), DUNDRUM-2 Comparison of security needs determined by multidisciplinary decision making (consultant psychiatrist, forensic social worker, and admission panel) and DUNDRUM-1 and DUNDRUM-2 scores. Details regarding scoring were not specified.	Comparison of DUNDRUM-1 mean scores indicated significantly higher mean scores for those: Offered admission to Broadmoor by the admission panel (mean = 3.40, SD = 0.39) compared to those not offered admission (mean = 3.33, SD = 0.37; ANOVA F = 4.21, P = 0.042). Recommended for admission by the assessing forensic psychiatrist (mean = 3.58, SD = 0.31) compared to those not recommended (mean = 3.34, SD = 0.38; ANOVA F = 22.83, df. = 1, p < 0.001) Recommended for admission by the forensic social worker (mean = 3.55, SD = 0.30) compared to those not recommended (mean = 3.33, SD = 0.42; ANOVA F = 16.21, df. = 1, p < 0.001). Comparison of DUNDRUM-1 scores across referral sources: Those referred from prison had significantly higher mean DUNDRUM-1 scores (mean = 3.59, SD = 0.33) compared to those referred from medium secure hospitals (mean = 3.27, SD = 0.32; ANOVA F = 25.28, df. = 1, p<0.001). Comparison of DUNDRUM-1 scores across pathways: Those referred to the personality disorder pathway had significantly higher mean DUNDRUM-1 scores (mean = 3.56, SD = 0.32) compared to those referred to the mental illness pathway (mean = 3.41, SD = 0.40; ANOVA F = 6.98, df. = 1, p = 0.009). DUNDRUM-2 There was no significant difference with regards to urgency of need for admission between the two pathways.	Not applicable.
	P. Habets; I. Jeandarme; H.G. Kennedy. 2020 Determining security level in forensic psychiatry: a tug of war between the DUNDRUM toolkit and the HoNOS-Secure.	Belgium	To examine the validity of the DUNDRUM-1 for distinguishing between required security level. To examine the relationship between DUNDRUM-2 scores and speed of diversion (length of stay in prison before diversion). To examine the inter-rater reliability of the DUNDRUM-1 and DUNDRUM-2	Design: Retrospective cohort Methodology: Four raters scored three measures including the DUNDRUM-1 and DUNDRUM-2 for a randomly selected sample of forensic psychiatric patients in prison. The raters were blind to the court decision when scoring the measures. The first author scored the whole sample and the other three raters each scored 10–15 patients. The DUNDRUM-1, DUNDRUM-2 were scored based on prison reports, clinical files of previous admissions and criminal responsibility evaluations. Court decisions were categorised on a scale of 1–4 (community to medium secure) based on DUNDRUM security levels. DUNDRUM-1 ratings were compared to court decisions. DUNDRUM-2 scores were compared to length of prison stay prior to diversion.	Referral source: Prison Admission setting: Psychiatric services (Community, open, low, medium, and high secure)	N = 100 A random sample of 100 male forensic psychiatric patients, selected using a random number generator, from a population of all 400 forensic psychiatric patients in prison. Overall sample characteristics: Male Mean age = 43.63 (SD = 11.6, range 23.2–71.9) 74% Belgian nationality, 44% psychotic disorder, 47% substance use disorder	DUNDRUM-1 (9-items), DUNDRUM-2 The external and predictive validity of the DUNDRUM-1: Comparison of court determined patient placement and scores on the DUNDRUM-1 ROC and whether the AUC indicated that the DUNDRUM-1 distinguished between security levels. Level of agreement between DUNDRUM-1 scores and court decisions. DUNDRUM-1 was scored on a 5-point scale: No security needed Open PICU/acute low Medium High Correlation between DUNDRUM-2 scores and length of time (in days) spent in prison. DUNDRUM-2: 0 (no current need for admission)- 4 (immediate admission) Inter-rater reliability of DUNDRUM-1 and DUNDRUM-2	DUNDRUM-1: Comparison of DUNDRUM-1 total scores showed a significant difference between scores across court decisions overall (Kruskall Wallis; p <0.05). ^b^ Post-hoc analyses indicated significantly lower scores for: Open security levels (mean = 14.4, SD = 3.4) v. medium security (mean = 22.0, SD = 4.3; p < 0.005) Open security levels v. high security (mean = 22.8, SD = 5.4; p < 0.005). Level of agreement 50% of DUNDRUM-1 medium-security decisions = high security court decisions 33% DUNDRUM-1 medium-security decisions = medium security court decisions 83% DUNDRUM-1 high-security decisions = high security court decisions Inter-rater reliability: Excellent for the total DUNDRUM-1 score and individual items were good to excellent except item 11: Legal process had average reliability. Three raters with DUNDRUM training had higher reliability (ICC = 0.90) compared to the rater without DUNDRUM training (ICC = 0.50). DUNDRUM-2: No DUNDRUM-2 scores indicated immediate need for admission, the average DUNDRUM-2 total score was 11.69 (SD = 2.3). No significant correlation between DUNDRUM-2 total scores and number of days in prison. Inter-rater reliability Overall average inter-rater reliability for the DUNDRUM-2 with three items having good reliability (Item 1: Current location, Item 3: Suicide prevention and Item 4: Humanitarian) and three items scoring low reliability (Item 2: Mental health, Item 5: Systemic and Item 6: Legal urgency).	DUNDRUM-1 predictive validity ROC: Predictive of high secure court decision (AUC = 0.63; p < 0.01) ^b^ Not predictive of medium-security court decisions. DUNDRUM-1 items predictive validity ROC: Two items were predictive of high-secure court decision Absconding/eloping (AUC = 0.62; p < 0.02) Legal process (AUC = 0.74; p < 0.01). ^b^
Cross- sectional	R. M. Jones; K. Patel; A. I. F. Simpson 2019 Assessment of need for inpatient treatment for mental disorder among female prisoners: a cross-sectional study of provincially detained women in Ontario	Canada	To examine the utility of the DUNDRUM-1 and DUNDRUM-2 for indicating admission to psychiatric hospital and distinguishing between required security level.	Design: Cross-sectional Methodology: Health care managers categorised mental health needs from ‘none’ to ‘very serious’, following initial screening. One of two forensic psychiatrists examined each file rated as ‘serious’ or ‘very serious’, alongside approximately 25% of files rated as ‘moderate’. A clinical assessment determined whether admission was necessary and whether this would be a high intensity admission. Each file was also rated on DUNDRUM-1 and DUNDRUM-2. 10 random files were rated by two clinicians. It was not stated whether the same clinicians conducted the initial assessment and DUNDRUM ratings or whether the DUNDRUM was completed blind to outcome. DUNDRUM ratings were compared across outcomes.	Referral source: Prison Admission setting: Secure hospital (general and high intensity Canadian security levels)	N = 66 On remand or sentenced prisoners in provincial jail in Ontario on 18^th^ July 2017. Sampled from a population of 643 prisoners, based on the assessed severity of mental health need. Mental health needs categories: N = 13 (2%) classified as ‘Very Serious’ N = 40 (6.2%) classified as ‘Serious’ (Three ‘Very Serious/Serious’ files were missing.) N = 16 classified as ‘Moderate’ (23.9% of 67 moderate cases) Overall sample characteristics: Female, Specific characteristics for the sample not reported.	DUNDRUM-1 (11 items), DUNDRUM- 2 The external and predictive validity of the DUNDRUM-1 and DUNDRUM-2: Comparison of need for admission and scores on the DUNDRUM-1 and DUNDRUM-2. ROC and whether the AUC indicated that the DUNDRUM-1 and DUNDRUM-2 distinguished those that require admission and between security levels. The relationship between DUNDRUM-1 and DUNDRUM-2 scores and need for admission. DUNDRUM-1 ratings on a 5-item scale ranged from 0–4 DUNDRUM-2 ratings on a 6-item scale.	DUNDRUM-1 Comparison of DUNDRUM-1 total score indicated significantly higher scores for those admitted to: High intensity secure hospital (mean = 25.1, SD = 4.7) v. general secure hospital admission, (mean = 17.5, SD = 6.9; t = 3.27, p = 0.003) General secure hospital admission v. no admission, (mean = 12.9, SD = 8.3; t = 2.10, p = 0.04) Need for admission was significantly related to DUNDRUM-1 total score, with likelihood of admission increasing as DUDNRUM-1 score increased (OR = 1.13, 95% CI 1.05–1.21, p < 0.001). DUNDRUM-2: Need for admission was significantly related to DUNDRUM-2 scores, with likelihood of admission increasing as DUNDRUM-2 scores increased (OR = 1.36, 95% CI 1.18–1.57, p < 0.001).	DUNDRUM-1 predictive validity ROC: Need for hospital admission v. no admission required, AUC = 0.75 (95% CI 0.63–0.88) High intensity secure hospital v. general secure hospital, AUC = 0.83, 95% CI 0.67–0.98) General secure hospital v no admission required, AUC = 0.68 (95% CI 0.53–0.82) Removing two items (seriousness of self-harm and immediacy of risk of suicide) from the total score increased the DUNDRUM-1 predictive validity slightly when comparing need for hospital admission v. no admission required (AUC = 0.82 95%, CI 0.71–93). DUNDRUM-2 predictive validity ROC: Need for hospital admission v. no admission required, AUC = 0.84 (95% CI 0.74–0.94) General secure hospital v no admission required, AUC = 0.81, (95% CI 0.67–0.94)
Prospective	C. Duggan; L. Mason; P. Banerjee; J. Milton 2007 Value of standard personality assessments in informing clinical decision–making in a medium secure unit	England	To examine the utility of standardised assessments (including the HCR-20) for indicating admission to a medium secure personality disorder unit.	Design: Prospective observational Methodology: Clinicians assessed referrals to the personality disorder service using several assessment measures, including the HCR-20. The current assessment procedure was then analysed by the researchers to determine how scores across measures were associated with admission.	Referral source: Not specified. Admission setting: Arnold Lodge medium secure personality disorder service	N = 89 Referrals for admission to a medium secure personality disorder service (1 February 1999–30 September 2005). N = 122 initially referred, N = 33 excluded for meeting exclusion criteria (IQ<80, history of psychosis, high psychopathy scores, a history of sadistic offending or lack of motivation). Overall sample characteristics: Male, Mean age = 27.9, High prevalence: major depression (47%), drug misuse (42%) and alcohol dependency (29%)	HCR-20 (version 2) Comparison of HCR-20 scores for those admitted and those not admitted. Higher scores on the HCR-20 represent a higher level of risk.	There were no significant differences in HCR-20 scores between those admitted and not admitted.	Not applicable.
	M. Freestone; D. Bull; R. Brown; N. Boast; F. Blazey; P. Gilluley 2015 Triage, decision-making and follow-up of patients referred to a UK forensic service: validation of the DUNDRUM toolkit	England	To examine the validity of the DUNDRUM-1 and DUNDRUM-2 for indicating admission to forensic secure services and distinguishing between required security level. To examine the internal consistency of the DUNDRUM-1.	Design Prospective Methodology: Forensic service referrals were allocated to a forensic psychiatrist that recorded information about the referral and made a recommendation to the admissions panel. The panel then used this information to score patients on the DUNDRUM-1 to determine whether admission was needed and DUNDRUM-2 (if accepted for admission) to determine priority for admission. The DUNDRUM was not scored blindly or independently to clinical judgement recommendations for placement. The DUNDRUM ratings were compared to decisions made using clinical judgement.	Referral source: Secure prison (Cat A or B; 42.6%), Open prison (Cat C or lower; 4.1%), Low secure/ open hospital (33.3%), Medium Secure hospital (3.1%), High Secure hospital (8.2%), Community (4.1%) Admission setting: PICU, East London NHS Foundation Trust Forensic service (Low and medium secure admission or high secure referral)	N = 195 N = 174 Male N = 21 Female All referrals for admission to a forensic service, with both a low and medium secure unit, between June 2011 and June 2013. Overall sample characteristics: 89% male, 11% female Mean age = 34.6 years 43.1% Black or Black British 50.3% diagnosed with schizophrenia.	DUNDRUM-1 (11-items), DUNDRUM-2 The external and predictive validity of the DUNDRUM-1: Comparison of scores on the DUNDRUM-1 and patient placement. ROC and whether the AUC indicated that the DUNDRUM-1 distinguished between those not admitted and those admitted to different security levels. Level of agreement between the DUNDRUM panel and assessing clinician. The relationship between the DUNDRUM-2 and time to admission. Details regarding scoring were not specified.	DUNDRUM-1 Comparison of DUNDRUM-1 total score across placement outcomes indicated that there was a significant effect of DUNDRUM-1 score on admission security level (ANOVA, F = 16.54, df = 4, p < .001). Post-hoc analyses observed significantly higher scores for those: Referred to high secure hospital (mean = 33.25, SD = 3.10) v. not admitted (mean = 22.56, SD = 7.54; mean difference = -10.69 p < 0.01) Referred to high secure hospital v. admitted to forensic low secure (mean = 19.53, SD = 4.84; mean difference = 13.72, p < 0.001) Admitted to medium secure (mean = 27.74, SD = 3.98) v. not admitted, (mean difference = -5.18, p < 0.001) Admitted to medium secure v. PICU, (mean = 19.31, SD = 4.84; mean difference = 8.43, p < 0.001) Admitted to medium secure v. forensic low secure, (mean difference = 8.21, p<0.001). Level of agreement The panel and clinician agreement was 66% (n = 107) Clinicians recommended admission more frequently than the DUNDRUM panel (n = 51; 31.5% of cases), with only four cases (2.5%) of the panel recommending admission when the clinician had not, (χ2(1) = 27.3, p < .001). DUNDRUM-2 There was not a significant relationship between DUNDRUM-2 scores and time to admission.	DUNDRUM-1 predictive validity (SMI sample) ROC: Those admitted to forensic medium secure, forensic low secure and PICU v. those not admitted AUC = 0.79 (95% CI 0.72–0.85), p<0.001. Differentiating between security levels ROC: PICU/Forensic low secure v. medium secure, AUC = 0.91 (95%CI 0.84–0.98, p < .001) Medium secure v. high secure hospital, AUC = 0.87 (95% CI 0.72–1.00, p = .040). There was not a significant improvement in AUC values by removing two items that were not predictive (Both if removing seriousness of self-harm (item 2) and immediacy of self-harm risk (item 4; p = 0.173) or institutional behaviour (item 10; p = 0.582). There was not a significant improvement in AUC values when DUNDRUM-1 and DUNDRUM-2 scores were combined, AUC = 0.74 (95% CI 0.55–0.94).
Prospective: Longer-term follow-up	G, Flynn., C, O’Neill., H.G, Kennedy 2011 DUNDRUM-2: Prospective validation of a structured professional judgment instrument assessing priority for admission from the waiting list for a forensic mental health hospital	Republic of Ireland	To examine the validity of the DUNDRUM-2 for prioritising patients on the waiting list for psychiatric hospital admission. To examine the relationship between the DUNDRUM-2, DUNDRUM-1, and admission need. To examine the inter-rater reliability of the DUNDRUM-2	Design: Prospective Methodology: One of the authors used pre-admission assessments to rate the DUNDRUM-1 and DUNDRUM-2 weekly for patients on the waiting list for psychiatric hospital admission (January-June 2010). 24 patients were independently rated by a second clinician. Those making decisions regarding admission priority were blind to the DUNDRUM ratings when deciding which admissions to prioritise. These patients’ outcomes were then observed for 6 months and compared to DUNDRUM-2 scores to identify whether the DUNDRUM-2 could prioritise the most urgent admissions.	Referral source: Prison (89%; On remand, 58%; sentenced, 31%), Less secure hospital (11%) Admission setting: Central Mental Hospital (Low, medium, high secure)	N = 65 N = 56 Male N = 10 Female ^e^ All those placed on the waiting list for admission to a psychiatric hospital over a six-month period (January–June 2010. Overall sample characteristics: Further characteristics not reported.	DUNDRUM-1 (11-items), DUNDRUM-2 The predictive validity of the DUNDRUM-2 for prioritising admission: Comparison of scores on the DUNDRUM-2 and patient outcomes with regards to admission. ROC and whether the AUC indicated that the DUNDRUM-2 was predictive of admission from the waiting list. Inter-rater reliability of the DUNDRUM-2 DUNDRUM-2 was assessed from 0 (no current need for admission) to 4 (immediate admission needed). DUNDRUM-1 Comparison of total scores on the DUNDRUM-1 and patient outcomes with regards to admission. ROC and whether the AUC indicated that the DUNDRUM-1 was predictive of admission from the waiting list. Comparison of scores on individual DUNDRUM-1 items and need for admission. ROC and whether the AUC indicated that the combined DUNDRUM-1 and DUNDRUM-2 scores were predictive of admission from the waiting list.	DUNDRUM-2 Comparison of DUNDRUM-2 scores when placed on the waiting list indicated scores were higher for those eventually admitted (mean = 12.5, SD = 3.2) v. not admitted (mean = 7.8, SD = 4.5), F = 11.0 (df = 1), p = 0.002. Comparison of DUNDRUM-2 scores when leaving the waiting list indicated scores were higher for those admitted (mean = 12.5, SD = 3.7) v. not admitted (mean = 5.3, SD = 3.4), F = 33.2 (df = 1), p<0.001. Inter-rater reliability No significant difference between mean scores for the two raters (t = -0.21, df = 23) Exact agreement across all 6 items was significant and ranged from 38%-100% (out of 24 cases): Item 1 (Location; 46% binomal exact probability, p < .004) Item 2 (Mental health; 42%, p < .013) Item 3 (Suicide prevention; 38%, p = .036) Item 4 (Humanitarian considerations; 50%, p < .001) Item 5 (Systemic considerations; 100%, p < .001) Item 6 (Legal urgency; 50% binomal exact probability, p < .001) DUNDRUM-1 Comparison of DUNDRUM-1 total scores when placed on the waiting list indicated scores were higher for those eventually admitted (mean = 25.3, SD = 6.5) v. not admitted (mean = 18.7, SD = 7.4), F = 4.4 (df = 1), p = 0.04. Comparison of DUNDRUM-1 scores when leaving the waiting list indicated scores were higher for those eventually admitted (mean = 25.3, SD = 6.6) v. not admitted (mean = 16.8, SD = 8.3), F = 7.6 (df = 1), p = 0.008.	DUNDRUM-2 ROC: DUNDRUM-2 scores were predictive of admission from the waiting list, both: Upon acceptance onto the waiting list AUC = 0.76 (95%CI 0.63–0.88) Upon leaving the waiting list AUC = 0.79 (95%CI 0.67–0.91) Comparison across locations ROC: DUNDRUM-2 scores were only predictive of admission for prisoners on remand: AUC = 0.91 (95%CI 0.81–1.00) DUNDRUM-1 DUNDRUM-1 scores were predictive of admission from the waiting list, both: Upon acceptance onto the waiting list AUC = 0.79 (95%CI 0.67–0.90) Upon leaving the waiting list AUC = 0.91 (95%CI 0.84–0.99). Comparisons across locations ROC: DUNDRUM-1 scores were predictive of admission for: Prisoners on remand AUC = 0.94 (95% CI 0.87–1.00) Sentenced prisoners AUC = 0.89 (95% CI 0.73–1.00) Patients in less secure hospitals AUC = 0.88 (95% CI 0.59–1.00) Combined DUNDRUM-1 and DUNDRUM-2 scores were predictive of admission from the waiting list, both: Upon acceptance onto the waiting list AUC = 0.79 (95% CI 0.68–0.90) Upon leaving the waiting list AUC = 0.87 (95% CI 0.78–0.96) Comparison across location: ROC Combined DUNDRUM-1 and DUNDRUM-2 scores were predictive of admission for: Remand prisoners AUC = 0.94 (95%CI 0.86–1.00) Sentenced prisoners AUC = 0.84 (95%CI 0.66–1.00) Patients in less secure hospitals AUC = 0.96 (95%CI 0.81–1.00)
	M, Davoren, S, O’Dwyer, Z, Abidin, L, Naughton, O, Gibbons, E, Doyle, K, McDonnell, S, Monk, & H. G., Kennedy 2012 Prospective in-patient cohort study of moves between levels of therapeutic security: The DUNDRUM-1 triage security, DUNDRUM-3 programme completion and DUNDRUM-4 recovery scales and the HCR-20	Republic of Ireland	To examine the validity of the DUNDRUM-1, DUNDRUM-3, DUNDRUM-4 and HCR-20 for distinguishing between those moved across security levels.	Design: Prospective in-patient cohort study (February 2010 –April 2011) Methodology: Two of the authors scored patient data gathered as part of a clinical audit using measures including the DUNDRUM-1, DUNDRUM-3, DUNDRUM-4 and the HCR-20. Patient movements between security levels were then observed and recorded as ‘positive’ or ‘negative moves’ for up to 13 months (Feb 2010- 31^st^ March 2011). Ratings across the measures were compared to patient movement.	Patient movement within Central Mental Hospital (Low, medium, high secure)	N = 86 All inpatients within the psychiatric hospital, across security levels, assessed between February and March 2010 (excluding seven patients that were either discharged or admitted late during the data collection period). Overall sample characteristics: Male, Mean age = 40.6 years (SD = 12.8), Most common primary diagnosis was schizophrenia (74%) followed by bi-polar affective disorder (10%), schizoaffective disorder (8%), major depressive disorder (3.5%) and intellectual disability (3.5%). Legal status was not guilty by reason of insanity (49%), prison to hospital transfer (26%), special transfer under the (civil) Mental Health Act (16%), unfit to stand trial (9%).	DUNDRUM-1 (11- items), DUNDRUM-3, DUNDRUM-4, HCR-20 (Version 2) The predictive validity of the DUNDRUM-1, DUNDRUM-3, DUNDRUM-4, and HCR-20 scales: Comparison of scores across the DUNDRUM-1, DUNDRUM-3, DUNDRUM-4 and HCR-20 scales and movement through levels of security over 13 months. ROC and whether the AUC indicated that these measures distinguished between security level movements over 13 months. The relationship between scores on each measure and either positive or negative moves No move = remaining at the same security level Positive move = move to lower security Negative move = move to higher security. The DUNDRUM-1 was scored on a 5-point scale from 0–4.	Comparison of DUNDRUM-1 total score, DUNDRUM-3, DUNDRUM-4, HCR-20-H and HCR-20-dynamic scores for patient movement (positive, negative and no moves) indicated that only scores on the DUNDRUM-1 and HCR-20 differed significantly across moves: DUNDRUM-1 (negative moves mean = 32.4, CI 29.3–35.7; no moves mean = 29.6, CI 28.5–30.8; positive moves mean = 26.6, CI 24.1–29.0), F = 4.1, p = 0.02 HCR-20-H (negative moves mean = 14.3, CI 11.8–16.8, no moves mean = 12.4, CI 11.7–13.1, positive moves mean = 15.2, CI 10.5–19.9), F = 3.5, p = 0.035 Positive moves Significantly lower mean DUNDRUM-1 total scores for those that had positive moves (mean = 26.6, SD = 3.7) compared to no move or negative move (mean = 29.9, SD = 4.8), F(df = 1) = 5.2 p = 0.026 Significantly higher mean HCR-20-H scores for those that had positive moves (mean = 15.2, SD = 6.9) compared to no move or negative move (mean = 12.6, SD = 3.0), F(df = 1) = 4.7, p = 0.034 Including location at baseline as a covariate led to significantly lower marginal mean scores for positive moves across: DUNDRUM-1 (Positive move marginal mean = 24.5, SE = 1.42, no move/negative move marginal mean = 30.3, SE = 0.51; F = 14.2, df = 1, p < 0.001) DUNDRUM-3 (Positive move marginal mean = 12.8, SE = 1.53, no move/ negative move marginal mean = 16.7, SE = 0.55; F = 5.4, p = 0.022) DUNDRUM-4 (Positive move marginal mean = 13.0, SE = 1.42, no move/negative move marginal mean = 16.7, SE = 0.51; F = 5.7, p = 0.02) HCR-20 ’C’ (Positive move marginal mean = 1.8, SE = 0.7, no move/ negative move marginal mean = 4.4 SE = 0.3; F = 11.2 p = 0.001). Including the HCR-20 dynamic (C+R) score as a covariate alongside location at baseline, the DUNDRUM-1 was the only measure with significantly lower scores fo positive (marginal mean = 23.9, SE = 1.5) compared to no move or negative move (marginal mean = 30.4, SE = 0.5; F = 15.9 df = 1 p < 0.001). Positive moves were influenced by: DUNDRUM-1 score (OR = 0.60, 95% CI 0.39–0.90, p = 0.014), Higher security need as indicated by a higher DUNDRUM-1 score associated with a reduced likelihood of a positive move. HCR-20-dynamic scale (clinical and risk scale combined) score (OR = 0.41, 95% CI 0.20–0.82, p = 0.012) Higher level of risk as indicated by a higher HCR-20 dynamic scale score associated with a reduced likelihood of a positive move. Negative moves Significantly higher mean DUNDRUM-1 scores for those that had negative moves (mean = 32.4, SD = 4.2) compared to no move or positive move (mean = 29.2, SD = 4.8), F(df 1) = 3.8 p = 0.053 Significantly higher mean DUNDRUM-4 scores for those that had negative moves (mean = 20.0, SD = 3.1) compared to no move or positive moves (mean = 15.8, SD = 6.1), F(df 1) = 4.2, p = 0.043 Significantly higher mean HCR-20-R scores for those that had negative moves (mean = 4.1, SD = 1.6) compared to no move or positive moves (mean = 2.5, SD = 2.2), F(df 1) = 4.7, p = 0.03 Significantly higher HCR-20 total scores for those that had negative moves (mean = 24.0, SD = 7.1) compared to no move or positive moves (mean = 19.1, SD = 6.9), F(df = 1) = 4.0, p = 0.048 Co-varying for location at baseline led to significantly higher marginal mean scores for negative moves across: DUNDRUM-1 (negative move marginal mean = 32.5, SE = 1.5, no move/positive move marginal mean = 29.2, SE = 0.5; F = 4.3, df = 1, p = 0.042) DUNDRUM-3 (negative move marginal mean = 19.8, SE = 1.5, no move/positive move marginal mean = 15.8, SE = 0.5; F = 6.2, df = 1, p = 0.015) DUNDRUM-4 (negative move marginal mean = 20.3, SE = 1.4, no move/positive move marginal mean = 15.8, SE = 0.5; F = 9.1, df = 1, p = 0.003) HCR-20 ’C’ (negative move marginal mean = 5.7, SE = 0.8, no move/positive move marginal mean = 3.9, SE = 0.3; F = 4.8, df = 1, p = 0.031) HCR-20 ’R’ (negative move marginal mean = 4.2, SE = 0.6, no move/positive move marginal mean = 2.5, SE = 0.2; F = 7.0, df = 1, p = 0.010) HCR-dynamic (’C’ + ’R’; negative move marginal mean = 9.9, SE = 1.3, no move/positive move marginal mean = 6.4, SE = 0.4; F = 6.8, df = 1, p = 0.011) HCR-20 total score (negative move marginal mean = 24.3, SE = 1.9, no move/positive move marginal mean = 19.1, SE = 0.6; F = 7.1, df = 1 p = 0.009) Including the HCR-20 dynamic score as a covariate alongside location at baseline, there were significantly higher scores for those that had negative moves for: DUNDRUM 1 (negative move marginal mean = 32.8, SE = 1.6, no move/positive move marginal mean = 29.2, SE = 0.5; F = 4.7, df = 1, p = 0.034) DUNDRUM-4 (negative move marginal mean = 19.2, SE = 1.4, no move/positive move marginal mean = 15.9, SE = 0.5; F = 5.2, df = 1, p = 0.025)^b^ Negative moves were influenced by: DUNDRUM-1 score (OR = 1.22, 95% CI 1.01–1.48, p = 0.038) Higher security need as indicated by a higher DUNDRUM-1 score associated with an increased likelihood of a negative move. HCR-20-dynamic score (odds ratio 1.27, 95% CI 1.05–1.54, p = 0.014) Higher level of risk as indicated by a higher HCR-20 dynamic (combined clinical and risk scale) score associated with an increased likelihood of a negative move.	Predictive validity ROC No measures were predictive of positive moves. Authors reported the DUNDRUM-1 as predictive due to a significant p value however AUC and CI were <0.5. Negative moves DUNDRUM-1, AUC = 0.70 (95% CI 0.54–0.86, p = 0.056) DUNDRUM-3 programme completion, AUC = 0.64 (95% CI 0.51–0.87, p = 0.163) DUNDRUM-4 recovery, AUC = 0.72, (95% CI 0.57–0.87, p = 0.032) HCR-20-dynamic score, AUC = 0.70, (95% CI 0.54–0.87, p = 0.048) (No other measures were predictive)
	M., Davoren, S., Hennessy, C., Conway, S., Marrinan, P., Gill, & H. G. Kennedy 2015 Recovery and concordance in a secure forensic psychiatry hospital–the self-rated DUNDRUM-3 programme completion and DUNDRUM-4 recovery scales	Republic of Ireland	To examine the validity of the DUNDRUM-3 and DUNDRUM-4 for distinguishing between those moved across security levels.	Design: Prospective cohort study (November 2011 –December 2012) Methodology: The first author completed the DUNDRUM-3 and DUNDRUM-4 for all patients, in February 2012, using data gathered as part of a clinical audit*. Ratings were conducted blind to outcome. The multidisciplinary team treating the patient also scored the HCR-20. Patient movements between security levels were then observed and recorded as ‘positive’ or ‘negative’ moves for 14-months (November 2011- December 2012). Ratings across the measures were compared to patient movement. *Patients also self-rated the DUNDRUM-3 and DUNDRUM-4 but this was not included for the purpose of the review.	Patient movement within Central Mental Hospital (Low, medium and high secure)	N = 97 N = 89 Male N = 8 Female All inpatients within the psychiatric hospital, across security levels. Overall sample characteristics: Mean age = 41 years (SD = 12.3) Most common diagnosis was schizophrenia (73%), followed by bi-polar affective disorder (11%), schizoaffective disorder (8%), recurrent depressive disorder severe with psychotic symptoms (4%) and intellectual disability (3%).	DUNDRUM-3, DUNDRUM-4, HCR-20 (Version 2) The validity of the DUNDRUM-3 and DUNDRUM-4: Comparison of DUNDRUM-3 and DUNDRUM-4 scores with outcome of movement through levels of security over 14 months. ROC and whether the AUC indicated that the self-rated and clinician-rated DUNDRUM-3 and DUNDRUM-4 and the HCR-20 distinguished between security level movements over 14 months. The relationship between scores on each measure and the influence on either positive or negative moves (regression). The internal consistency of both self-rated and clinician-rated DUNDRUM-3 and DUNDRUM-4.	Positive moves Those that had positive moves had significantly lower mean scores on the DUNDRUM-3 (mean = 1.9, SD = 0.9) compared to those that did not have positive moves (mean = 2.6, SD = 0.9), F = 9.4, p = 0.003 Those that had positive moves also had significantly lower mean scores on the DUNDRUM-4 (mean = 2.2, SD = 0.9) compared to those that did not have positive moves (mean = 2.8, SD = 0.9), F = 6.7, p = 0.012. Positive moves were influenced by: DUNDRUM-3 score (OR = 0.88, 95% CI 0.81–0.97, p = 0.006, higher score = less progress and less likely positive move) HCR-20-dynamic score (Clinical & Risk scale combined) (OR = 0.77, 95% CI 0.66–0.90, p = 0.001) Negative moves Those that had negative moves had significantly higher mean scores on the DUNDRUM-3 (mean = 2.9, SD = 0.8) compared to those that did not have negative moves (mean = 2.1, SD = 0.8), F = 5.4, p = 0.023 Those that had negative moves also had significantly higher mean scores on the DUNDRUM-4 (mean = 3.3, SD = 0.6) compared to those that did not have negative moves (mean = 2.4, SD = 0.9), F = 6.9, p = 0.011. DUNDRUM-1 score had no effect on these models.	Predictive validity ROC DUNDRUM-3 Positive moves (AUC = 0.72, 95% CI 0.59–0.85, p = 0.005) Negative moves (AUC = 0.76, 95% CI 0.59–0.93, p = 0.019) DUNDRUM-4 Positive moves (AUC = 0.70, 95% CI 0.56–0.83, p = 0.011). Negative moves (AUC = 0.78, 95% CI 0.64–0.93, p = 0.010 HCR-20 dynamic (C+R) Positive moves (AUC = 0.79, 95% CI 0.68–0.91, p < 0.001) Negative moves (AUC = 0.71, 95% CI 0.55–0.86, p = 0.063) DUNDRUM-1 was not predictive of either positive or negative moves.
	S., McCullough, C., Stanley, H., Smith, M., Scott, M., Karia, B., Ndubuisi,. . . & M, Davoren 2020, Outcome measures of risk and recovery in Broadmoor High Secure Forensic Hospital: stratification of care pathways and moves to medium secure hospitals.	England	To examine whether DUNDRUM-3, DUNDRUM-4 and HCR-20 scores were different for those moved from high secure to medium secure services.	Design: Prospective cohort study (May 2016- June 2017) Methodology: The research team scored all Broadmoor patients using the DUNDRUM-3 and DUNDRUM-4 alongside standard assessments made by clinicians on the HCR-20. While only the researchers had access to the scores on the DUNDRUM-3 and DUNDRUM-4, the HCR-20 scores were available to clinicians as part of the standard assessment process. Patient outcomes regarding movement to medium secure were observed for a period of 13-months. Ratings across the measures were compared for those that moved to medium secure services and those that did not.	Initial setting: Broadmoor high secure hospital (62% mental illness pathway, 38% personality disorder pathway) Step-down setting: Medium secure services	N = 142 All Broadmoor high secure hospital inpatients with sufficient data at the beginning of the study (May and June, 2016) to accurately rate the scales (N = 50 excluded due to insufficient data). Overall sample characteristics: Male, Mean age = 39.5 years, Most common diagnosis of schizophrenia (56.8%), followed by dissocial personality disorder (15.3%), schizoaffective disorder (11.4%) and neurocognitive disorders (6.8%) (patients could have comorbid diagnoses).	DUNDRUM-3, DUNDRUM-4, HCR-20 (Version 3) Comparison of scores on the DUNDRUM-3, DUNDRUM-4 and HCR-20 and movements to lower security levels across 13 months.	Patients granted trial leave to medium secure units during 13-month follow up were found to have significantly lower scores on: HCR-20-V3 Clinical scale (trial leave mean = 4.5, SD = 2.8; no trial leave mean = 7.0, SD = 2.6), F = 30.99, p<0.001 HCR-20-V3 Risk (future) scale (trial leave mean = 5.9, SD = 2.2; no trial leave mean = 7.1, SD = 2.4), F = 22.88, p<0.001 ^c^ HCR-20-V3 dynamic (current and future) scale (trial leave mean = 10.5, SD = 4.8; no trial leave mean = 14.1, SD = 4.7), F = 33.20, p<0.001 DUNDRUM-3 (trial leave mean = 22.7, SD = 3.3; no trial leave mean = 24.4, SD = 2.5), F = 9.24, p<0.001 DUNDRUM-4 (trial leave mean = 22.0, SD = 2.8; no trial leave mean = 23.8, SD = 2.1), F = 6.86, p = 0.001 Scores on the HCR-20-Historical scale were not significantly associated with step-down moves to medium secure.	Not applicable.

Please note. Throughout the table only aims, methodology and findings relevant to the review research question were included so while further exploration of measures such as cross-validation, internal consistency and factor analysis were conducted in some studies, this information has not been extracted for purposes of the review. Item-level findings can be found in S3 Table and S1 Text.

*Note* ^*a*.^ All figures are reported to 2 decimal places (where provided in the original article) aside from p values reported up to 3 decimal places.

*Note* ^*b*^. All statistics not reported in the paper.

*Note* ^*c*.^ The statistics presented in the table did not match those reported in the text, the statistics reported in this review are those presented in the table.

*Note* ^*d*^. Values (including means, AUCs and 95% CIs) are approximate due to extraction from a figure.

*Note* ^*e*^. The sample sizes do not add up.

### Study characteristics

#### Study background

Articles identified in this review were conducted between 2007 and 2020; eight were conducted less than 10 years ago. The research was conducted across five countries: England (n = 4) [39, 41, 45, 50], Wales (n = 1) [43], The Republic of Ireland (n = 4) [40, 46–48], Belgium (n = 2) [42, 49] and Canada (n = 1) [43].

#### Aims

Most studies aimed to determine the predictive validity of the studied SPJ guidance (N = 9) [40–44, 46–49]. The remaining three focused on the value and utility of the chosen SPJ guidance for assessing security needs, both for admission and step-down movements [39, 45, 50]. Three studies also examined the inter-rater reliability of the studied SPJ guidance [40, 48, 49]. Not all aims, measures and findings presented in each study are discussed in this review. For more information on the research conducted more widely, please refer to the original papers. The text below (alongside Table 3) presents findings related only to the SPJ approach indicating admission and assessing security needs, including movements between security.

#### Study design and methodology

Study designs were retrospective cohort (n = 5), cross-sectional (n = 1), and prospective-cohort designs (n = 6, including four that we categorised as ‘prospective: longer-term follow-up’). Prospective studies were subdivided due to two measuring outcomes immediately following assessment [39, 41], while the remaining four measured outcomes over a longer follow-up period of 6–14 months [46–48, 50]. Due to the variability in follow-up period across prospective studies, these subcategories were created to increase ease of comparison and acknowledge variation in study design across prospective studies.

The retrospective cohort studies compared the admission recommendation based on the usual decision-making process, such as a court decision or clinical assessment, to ratings on the studied SPJ guidance [40, 42, 44, 45, 49]. The methodology of the cross-sectional study was similar. The studied SPJ guidance was completed by a clinician and compared to real-time clinical assessment decisions regarding the need for admission and security level [43]. It is unclear, based on the information provided, whether the clinical assessment and use of SPJ guidance were independent [43]. A key difference in this study from other designs is pre-assessment screening of mental health needs, with only those rated as having more severe mental health needs being included for further clinical and SPJ assessment [43].

One prospective study compared a clinical judgement recommendation to ratings on a particular set of SPJ guidance tallied into a score. Forensic service referrals were evaluated by a psychiatrist, with a recommendation submitted to a panel that then completed the DUNDRUM-1 for all service users and DUNDRUM-2 for service users that were identified as needing admission. DUNDRUM outcome and clinician decisions were then compared [41]. It is unclear whether the panel were aware of the initial clinical recommendation prior to rating the SPJ guidance. The second prospective study did not share this methodology as clinicians assessed referrals to a medium secure personality disorder service using a range of assessment guidelines, with researchers then comparing scores for service users that were and were not admitted [39].

Three of the four longer-term follow-up studies utilised similar methodologies, rating service users based on SPJ-informed guidance and then observing service user movement over 13 or 14 months [46, 47, 50]. Two focused on positive (movements to a lower security level) or negative (movements to a higher security level) moves [46, 47] while the third [50] focused specifically on movement from high to medium secure services. The remaining longer-term follow-up study differed slightly due to the shorter follow-up duration and sample not yet admitted [48]. One of the authors scored the SPJ guidance weekly for those on the waiting list for admission, these scores were then compared to outcomes in terms of movement from the waiting list over a 6-month period [48].

#### Sample

Sample sizes ranged from 50 to 316 participants, with over half of samples including 100 participants or more [40–42, 44, 45, 49, 50]. Samples were predominantly male, with seven focusing on all-male samples [39, 40, 42, 45, 46, 49, 50]. One study included a female-only sample [43] and three included both male and female participants [41, 47, 48]. One study did not report these sample characteristics [44]. The mean age of participants was reported across eight studies, ranging from 27.9 years to 43.6 years with an overall mean age of 37.9 years [39, 41, 42, 45–47, 49, 50].

#### Admission setting and referral source

The most frequently observed admission setting was medium security [n = 10; 39–42, 44, 46–50] followed by low security [n = 7; 40, 41, 44, 46–49] and high security [n = 7, 40, 41, 45–49,]. Canadian services included were described as ‘general secure’ or ‘high intensity secure’ hospitals [43]. Non-secure setting comparisons, including open and community settings were only included to determine the ability of an SPJ approach to distinguish between the need for secure or non-secure environments.

Ten studies reported referral source, including those that were observing movement across security levels within the same hospital [40–43, 45–50]. Three studies focused exclusively on referrals from prison [40, 43, 49], and one on service users found not guilty by reason of insanity and receiving a treatment order referred by courts [42]. The remaining studies observed referrals from multiple sources including prisons [41, 45, 48], high secure [41], medium secure [41, 45], less secure hospital [48], low secure/open settings [41] and the community [41]. Three studies observed service users already receiving treatment in secure services and examined step-up or step-down movement across levels of secure services within the same hospital [46, 47] or specifically moving from high secure to medium secure [50].

### SPJ guidelines

The most frequently studied SPJ guidance was the DUNDRUM-1 triage security measure [n = 9; 40–46, 48, 49]. The DUNDRUM-1 intends to guide decision-making regarding the most appropriate security level for someone requiring psychiatric hospital admission [25]. The studies in this review explored the ability of the DUNDRUM-1 to indicate need for admission and/or differentiate between the security level required. The DUNDRUM-1 was initially developed with eleven items and all eleven items were used across six studies [40, 41, 43, 44, 46, 48]. Three studies [42, 45, 49] used the adapted nine-item scale (exclusion of item 2: Seriousness of Self-harm and item 4: Immediacy of self-harm) recommended due to two items found in earlier studies to correspond less with service user placement than the remaining nine [25]. The authors of the DUNDRUM-1 recommend calculating a mean score for each service user to indicate the most appropriate security level (mean >3 = high security, >2 = medium security, >1 = low security, 0–1 = open hospital or community) [25]. While calculating mean scores for each service user (before calculating an overall sample mean) is recommended, only two studies used the mean score for each service user across nine items [42, 45] with seven using service user total scores of eleven [40, 41, 43, 44, 46, 48] or nine items [49]. All findings should be read within this context and throughout the review findings *DUNDRUM-1 score* may refer to the mean or total score, depending on study.

The DUNDRUM-2 was included in five studies [41, 43, 45, 48, 49]. The DUNDRUM-2 intends to guide decision making regarding priority for admission to secure services from a waiting list [25]. The studies in this review explored the ability of the DUNDRUM-2 to indicate priority for admission alongside need for admission.

Five studies also included the HCR-20 [39, 40, 46, 47, 50]. While the HCR-20 [Currently on Version 3] was initially developed to support risk assessment and risk management decisions [52], the studies explored the ability of the HCR-20 to indicate a need for admission or distinguish between service users moved across security levels. Three studies used the HCR-20 (Version 2) [39, 46, 47] while one used the HCR-20 (Version 3) [50].

The DUNDRUM-3 and DUNDRUM-4 were the focus of three studies [46, 47, 50]. The DUNDRUM-3 and DUNDRUM-4 were developed to be used together and support decisions on service users’ readiness to move to a lower security level [25]. The DUNDRUM-3 is specifically intended to indicate whether treatment programmes have been completed to reduce risk while the DUNDRUM-4 focuses on recovery [25]. The studies in this review explored how ratings on the DUNDRUM-3 and DUNDRUM-4 may distinguish between service users either moved to higher or lower security levels or not moved between security levels. The remaining synthesis is presented according to SPJ guideline to allow for direct comparison of findings. Item level findings are presented as supplementary information (S3 Table and S1 Text).

### DUNDRUM-1

*Need for admission*. Five studies examined how the need for admission related to DUMDRUM-1 outcome and whether the DUNDRUM-1 can distinguish between those requiring or not requiring admission, as assessed by clinicians [40, 41, 43, 45, 48]. It was observed that those admitted to high secure hospital (F = 4.21, p = 0.042 [45]; p<0.001 [41]), medium secure services (p<0.001 [41]) and ‘general security’ hospitals (t = 2.10, p = 0.04 [43]) had significantly higher DUNDRUM-1 scores than those not admitted. Similarly, those recommended for admission by assessing professionals had significantly higher DUNDRUM-1 scores (Forensic psychiatrist: mean = 3.58, SD = 0.31; Forensic social worker: mean = 3.55, SD = 0.30) compared to those not recommended (Forensic psychiatrist: mean = 3.34, SD = 0.38; ANOVA F = 22.83, df. = 1, p < 0.001; Forensic social worker: mean = 3.33, SD = 0.42; ANOVA F = 16.21, df. = 1, p < 0.001) [45]. Likewise, those admitted to secure services were observed to have significantly higher DUNDRUM-1 scores when initially placed on a waiting list (F = 4.4, df = 1. P = 0.04) and when leaving the waiting list (F = 7.6, df = 1, p = 0.008), compared to those not requiring admission [48]. Prisoners requiring a full psychiatric assessment that were subsequently admitted to any available psychiatric hospital (including but not limited to secure care; mean = 15.77, SD = 5.33) had significantly higher DUNDRUM-1 scores compared to those not admitted and receiving psychiatric follow-up (mean = 4.14, SD = 4.57) or discharged to the prison GP (mean = 0.21, SD = 0.79; Bonferroni statistics not provided) [40]. Likelihood of admission was also observed to increase as DUNDRUM-1 scores increased (Total: OR = 1.13, 95%CI 1.05–1.21, p<0.001) [43].

*Required security level*. Six studies examined how DUNDRUM-1 scores may be able to differentiate between those requiring varying levels of security [40–44, 49]. The first DUNDRUM-1 study observed that, among those diverted from prison to psychiatric hospital, there were significantly higher DUNDRUM-1 scores for those admitted to higher levels of security, however post-hoc Bonferroni statistics were not reported: Medium and high secure mean = 22.87 (SD = 4.56); Psychiatric intensive care unit [PICU] mean = 15.81 (SD = 4.39); Open wards mean = 10.74 (SD = 3.26) [40]. Five studies further examined the ability of the DUNDRUM-1 to differentiate between required security level in settings other than where the DUNDRUM-1 was initially developed [41–44, 49]. Initial analyses indicated that there were significant differences in DUNDRUM-1 scores across security levels (H = 0.737, df = 2, p<0.000 [44]; p<0.05 [42]; p<0.05 [49]), however did not indicate between which security level these differences were present. Further post-hoc analyses indicated that those placed in low secure settings (mean = 23.86) had significantly higher DUNDRUM-1 scores compared to those placed in open settings (mean = 20.00; p<0.050 [44]) and significantly lower DUNDRUM-1 scores compared to those placed in medium security settings (p<0.001 [41]; mean = 29.64 p<0.005 [44]) and high security settings (p<0.001 [41]). Service users placed within medium security settings had significantly higher DUNDRUM-1 scores compared to those needing open settings (p<0.005 [44]; p<0.005 [49]) and PICU (p<0.001 [41]) and significantly lower DUNDRUM-1 scores than those needing high security (p<0.01 [42]). Scores for service users needing high security were also significantly higher than those needing open settings (p<0.01 [42] p<0.005 [49]). Those requiring high intensity hospital in Canada also had significantly higher DUNDRUM-1 scores than those admitted to general security hospital (t = 3.27, p = 0.003) [43]. In the context of a Belgian forensic psychiatric setting, court recommendations for higher security levels were significantly, although weakly associated with higher DUNDRUM-1 mean score (r_s_ = 0.25, p<0.05) [42].

*Service user movement between security levels*. One study examined how DUNDRUM-1 scores may be able to differentiate between those moved, or not, between security levels [46]. Higher DUNDRUM-1 scores were associated with decreased likelihood of movement to lower security (OR = 0.60 95% CI 0.39–0.90, p = 0.014) and increased likelihood of movement to higher security (OR = 1.22, 95%CI 1.01–1.48, p = 0.038) [46]. Scores were significantly higher for those moved to higher security levels (F = 3.8, df = 1, p = 0.053) and significantly lower for those moved to lower security levels (F = 5.2, df = 1, p = 0.026) [46].

*Relationship between clinical or court decision and DUNDRUM-1 scores*. Two studies examined the percentage of court decisions and DUNDRUM-1 scores in agreement regarding the required security level [42, 49]. One study observed that a greater percentage of court decisions recommended high security compared to the DUNDRUM-1, with 50% of cases in which the DUNDRUM-1 recommended medium security corresponding to a high-security court decision [49]. While 83% of DUNDRUM-1 scores indicating high security corresponded to high-security court decision, only 33% of scores indicating medium security corresponded to medium-security court decision [49]. This was supported by another study observing that overall agreement between court recommendations and DUNDRUM-1 score (40%) was poor (k = 0.06, p = 0.15), with court recommendations indicating higher security more frequently than the DUNDRUM-1 score (45.5%) [42].

One study compared (non-SPJ) clinical decision-making with security levels indicated by the DUNDRUM-1 [41]. Comparison of a clinical recommendation by a forensic psychiatrist with a panel-scored DUNDRUM-1 indicated agreement in 66% of cases (n = 107), although it should be noted that the DUNDRUM-1 was scored by the panel based on information provided by the clinician [41]. Clinicians recommended admission significantly more frequently than the panel using the DUNDRUM-1 (n = 51; 31.5% of cases), with only four cases (2.5%) where the panel recommended admission when the clinician had not, (χ2(1) = 27.3, p < .001;) [41].

*Predictive validity of the DUNDRUM-1*. Eight studies assessed the predictive validity of the DUNDRUM-1 [40–44, 46, 48, 49]; six observed admissions to secure services as the outcome, one observed movement between security levels [46] while another study examined the predictive validity of the DUNDRUM-1 for admission from the waiting list [48]. The predictive validity of the DUNDRUM-1 was measured using receiver operating characteristics (ROC), specifically the Area under the Curve (AUC). Four studies found that the DUNDRUM-1 was predictive for indicating admission to secure services, with the AUC ranging from 0.75 (95%CI 0.63–0.88) to 0.98 (95% CI 0.97–0.98) [40, 41, 43, 44]. The highest predictive accuracy for admission when compared to no admission was identified in the first DUNDRUM-1 validation study among a sample identified for full psychiatric assessment (AUC = 0.98, 95%CI 0.97–0.98) [40].

The findings were variable regarding whether the DUNDRUM-1 was predictive for decisions to admit to low, medium, or high secure services. It was found to be predictive of medium or high secure admission when compared to PICU (AUC = 0.87, 95%CI 0.78–0.95 [40]), alongside distinguishing between medium secure and high secure admission (AUC = 0.87, 95%CI 0.72–1.00 [41]) and medium secure and PICU/low secure admission (AUC = 0.91, 95%CI 0.84–0.98 [41]). Two studies found the DUNDRUM-1 to be predictive of high secure admission (AUC = 0.63, p<0.01- AUC 0.70, p<0.05 [42, 49]) and able to distinguish between high intensity and general hospital admission (AUC = 0.83, 95%CI 0.67–0.98 [43]).

The DUNDRUM-1 was also predictive of admission from the waiting list, distinguishing between those admitted or not admitted, both based on scores when services were placed on the waiting list (AUC = 0.79, 95%CI 0.67–0.90) and when they were removed from the waiting list (AUC = 0.91, 95%CI 0.84–0.99) [48]. Location upon referral did not influence this predictive accuracy as the DUNDRUM-1 was predictive of admission for those referred while on remand (AUC = 0.94, 95%CI 0.87–1.00), sentenced (AUC = 0.89, 95%CI 0.73–1.00) or in less secure hospitals (AUC = 0.88, 95%CI 0.59–1.00) [48]. With regards to movement between security levels, the DUNDRUM-1 was only predictive of ‘negative moves’ to higher security levels (AUC = 0.70, 95%CI 0.54–0.86) [46]. Comparisons for non-secure environments are presented in Table 3.

*Inter-rater reliability*. Two studies measured the inter-rater reliability of the DUNDRUM-1 [40, 49]. One study reported that 18 cases were independently rated by two clinicians [40]. Cohen’s Kappa was calculated for 7/11 items and was greater than 0.85 (p<0.001) for these items, indicating strong agreement between the two clinicians [40, 53]. There was also a significant correlation between DUNDRUM-1 scores of both clinicians for all 11 items (correlation greater than rs = 0.75, p<0.001 for each item) and the total scores (for 11 items) of both clinicians were significantly correlated (rs = 0.96, p<0.001) [40]. The second study reported excellent inter-rater reliability for the total DUNDRUM-1 score, although inter-rater reliability was better for those that had received DUNDRUM training (Intraclass correlation coefficient [ICC] = 0.90) compared to the rater that had not (ICC = 0.50) [49].

### DUNDRUM-2

*Priority for admission*. Four studies examined whether the DUNDRUM-2 could prioritise those most urgently in need of admission [41, 45, 48, 49]. The initial validation study observed that the DUNDRUM-2 was able to prioritise those admitted from the waiting list over those not admitted [48]. Those admitted scored as higher priority on the DUNDRUM-2, with significantly higher scores compared to those not admitted, both when placed on the waiting list (F = 11.00, df = 1, p = 0.002) and when leaving the waiting list (F = 33.2, df = 1, p<0.001) [48].

Three studies examined priority for admission by investigating the relationship between how urgently a service user was admitted to secure services and DUNDRUM-2 score [41, 45, 49]. Scores were not significantly associated with time to admission [41] or the speed of diversion from prison [49]. In a high secure setting, those admitted to two different treatment pathways (mental illness or personality disorder) did not differ in urgency of need for admission, based on DUNDRUM-2 score [45].

*Need for admission*. Research in a forensic psychiatric setting in Canada investigated what they termed “need for admission” based on the DUNDRUM-2 score and observed a significant relationship, with higher scores related to increased likelihood of admission (OR = 1.36, 95%CI 1.18–1.57, p<0.001) [43].

*Predictive validity of the DUNDRUM-2*. Two studies measured the predictive validity of the DUNDRUM-2 for indicating admission [43, 48]. The DUNDRUM-2 was able to differentiate between those that were or were not admitted from the waiting list, including at two time points of service users being placed on the waiting list for admission (AUC = 0.76, 95%CI 0.63–0.88) and when leaving the waiting list (AUC = 0.79, 95%CI 0.67–0.91) [48]. When comparing groups on the waiting list, scores were predictive of admission for those on remand (AUC = 0.91, 95%CI 0.81–1.00) but not those sentenced or in a less secure hospital setting [48]. When the DUNDRUM-1 was added as a covariate, the model remained predictive both when service users were accepted onto the waiting list (Combined AUC = 0.79, 95%CI 0.68–0.90 compared to DUNDRUM-2 AUC = 0.76 95%CI 0.63–0.88) and when leaving the waiting list (Combined AUC = 0.87, 95%CI 0.78–0.96 compared to DUNDRUM-2 AUC = 0.79 95%CI 0.67–0.91) [48]. With regards to predictive validity for admission to a specific security level, the DUNDRUM-2 was predictive of need for admission compared to no admission required (AUC = 0.84, 95% CI 0.74–0.94) and Canadian general security hospital admission compared to no admission required (AUC = 0.81, 95%CI 0.67–0.94) however was not predictive of high intensity admission [43].

*Inter-rater reliability*. Inter-rater reliability of the DUNDRUM-2 was examined across two studies, with one study reporting no significant difference in mean scores between raters and exact agreement was significant (p<0.05) across all items (Agreement ranging from 38%, p = 0.036 for Item 3: Suicide Prevention– 100%, p<0.001 for Item 5: Systemic) [48]. The second study reported overall average reliability (Three items with good reliability included Item 1: Current location, Item 3: Suicide prevention and Item 4: Humanitarian and three items scoring low reliability included Item 2: Mental health, Item 5: Systemic and Item 6: Legal urgency) [49].

### HCR-20, versions 2 and 3

*Need for admission*. One study evaluated the extent to which differences in HCR-20 (Version 2) scores could distinguish between those admitted or not admitted [39]. Those admitted to a medium secure personality disorder service did not significantly differ in HCR-20 total scores compared to those not admitted [39].

*Service user movement between security levels*. Two studies examined the extent to which the HCR-20 could differentiate between service users moved to higher security levels, lower security levels or remaining at the same security level [46, 47]. Service users moved to higher security levels had significantly higher total HCR-20 scores (F = 4.0, df = 1, p = 0.048) and this difference remained significant when location at baseline was included as a covariate (F = 7.1, df = 1, p = 0.009) [46].

When comparing scores on individual subscales, higher scores on the HCR-20 Dynamic scale (Combined Clinical and Risk scales) were observed to be associated with a reduced likelihood of movement to lower security (OR ranging from 0.41 95%CI 0.204–0.821, p = 0.012 [46] to 0.77 [95%CI 0.66–0.90, p = 0.001 [47]) and increased likelihood of movement to higher security (OR = 1.27 [95%CI 1.05–1.54, p = 0.014 [46]). Those moved from high security to medium security had significantly lower scores on the HCR-20 Dynamic scale (Combined Clinical and Risk scales) than those not moved (F = 33.199, p<0.001) [50]. With location at baseline included as a covariate, the HCR-20 Dynamic scores were significantly higher for those moved to a higher security level compared to those not moved or moved to a lower security level (F = 6.8, df = 1, p = 0.011) [46]. When the components of the HCR-20 Dynamic scale, the Clinical (HCR-20-C) and Risk (HCR-20-R) scales, were evaluated separately, those moved from high to medium security scored significantly lower on the HCR-20-C scale compared to those remaining in high security (F = 30.993, p<0.001) [50]. Similarly, the HCR-20-C score was significantly lower for step-down movements to lower security compared to no move or step-up movement to higher security (F = 11.2, p = 0.001) and significantly higher for step-up movements to higher security compared to no move or step-down movement to lower security (F = 4.8, df = 1, p = 0.031), although these differences were only present when location at baseline was included as a covariate [46].

The HCR-20-R score was significantly higher for those moved to higher security levels compared to no move or step-down movement to lower security (F = 4.7, df = 1, p = 0.03) [46]. This difference remained significant with location at baseline as a covariate (F = 6.9, df = 1, p = 0.01) [46]. Those moved from high to medium security scored significantly lower on the HCR-20 Future scale score than those remaining in high security (F = 22.88, p<0.001) [50]. One study observed that the HCR-20 Historical (HCR-20-H) score was significantly higher for those moved to lower security levels compared to those not moved or moved to higher security (F = 4.7, df = 1, p = 0.034), although this difference was no longer significant when location at baseline was included as a covariate [46].

*The predictive validity of the HCR-20 for access assessments*. The two studies examining the predictive validity of the HCR-20 used service user movements to higher or lower security levels as the outcome measure [46, 47]. The HCR-20 Dynamic scale (Combined clinical and risk scale) was found to be predictive of positive moves to lower security levels (AUC = 0.791, 95%CI 0.68–0.91, p<0.001 [47]) and negative moves to higher security levels (AUC between 0.70, 95%CI 0.54–0.87, p = 0.048 and 0.71, 95% CI 0.55–0.86, p = 0.063 [46, 47]). The HCR-20 total score and remaining sub-scales (the historical, clinical and risk management subscales separately) were not significantly predictive of service user movements.

### DUNDRUM-3 and DUNDRUM-4

*Service user movement between security levels*. Three studies examined how DUNDRUM-3 and DUNDRUM-4 scores may differentiate between those moved or not between security levels [46, 47, 50]. While all three studies focused on service user movement as the outcome, one study specifically focused on movement from high security to medium security, with those moved to medium-security having significantly lower DUNDRUM-3 (F = 9.24, p<0.001) and DUNDRUM-4 scores compared to those remaining in high security (F = 6.86, p = 0.001) [50].

Two studies compared movements to both higher (step-up) and lower (step-down) security levels [46, 47]. The more recent study observed that DUNDRUM-3 scores were significantly higher for those moved to higher security (F = 5.4, p = 0.023) and significantly lower for those moved to lower security (F = 9.4, p = 0.003) compared to those not moved [47]. Higher DUNDRUM-3 scores were associated with reduced progress and decreased likelihood of movement to a lower security level (OR = 0.88, 95%CI = 0.81–0.97) [47]. Meanwhile, the earlier study observed that DUNDRUM-3 scores were only significantly higher for those moved to higher security compared to no move or move to lower security (F = 6.2, df = 1, p = 0.015) and significantly lower for those moved to lower security compared to no move or move to higher security (F = 5.4, df = 1, p = 0.022) when location at baseline was included as a covariate [46].

Both studies that measured step-up and step-down movements observed that those moved to higher security levels had significantly higher scores on the DUNDRUM-4 compared to those that were not moved to higher security (F = 6.9, p = 0.011 [47]) and those not moved or moved to lower security (F = 4.2, df = 1, p = 0.043 [46]). Service users moved to lower security levels also had significantly lower DUNDRUM-4 scores compared to those that were not moved to lower security (F = 6.7, p = 0.012) [47]. However, in one study lower scores for those moved to lower security levels compared to those not moved or moved to higher security levels were only observed when location at baseline was included as a covariate (F = 5.7, p = 0.02) [46].

*Predictive validity of the DUNDRUM-3 and DUNDRUM-4*. Two studies examined whether the DUNDRUM-3 and DUNDRUM-4 were predictive of service user movements across security levels [46, 47]. The DUNDRUM-3 and DUNDRUM-4 were found to be predictive of moves to higher security levels in both studies [46, 47]. DUNDRUM-3 AUC ranged from 0.64 (95%CI 0.51–0.87) to 0.76 (95%CI 0.59–0.93) while DUNDRUM-4 AUC ranged from 0.72 (95%CI 0.57–0.87) to 0.78 (95%CI 0.64–0.93) [46, 47]. Both measures were also found to be predictive of moves to lower security levels in one of the two studies (DUNDRUM-3, AUC = 0.72, 95%CI 0.59–0.85 and DUNDRUM-4, AUC = 0.70, 95%CI 0.56–0.83) [47].

## Discussion

This review aimed to address a gap in the evidence base regarding the use of the SPJ approach for adult SPS admission assessments. The SPJ approach was developed within the risk assessment and management literature to promote systemisation and consistency whilst facilitating discretion [23]. However, as highlighted in this review, SPJ guidance developed for the purpose of violence risk assessment, such as the HCR-20, has been applied to admission assessments alongside new guidelines, such as the DUNDRUM toolkit, specifically developed for the purpose of admission assessment. Twelve studies were reviewed evaluating five SPJ-informed guidelines [DUNDRUM-1, DUNDRUM-2, DUNDRUM-3, DUNDRUM-4, and HCR-20] for the purpose of initial admission assessment or assessment for movement between security levels. The current evidence was summarised in terms of the predictive validity and utility of SPJ-informed guidelines for admission assessments to examine the applicability in this context.

The overall pattern emerging across studies was that higher scores across the different sets of SPJ guidance were associated with an increased likelihood of admission, specifically admission to higher security wards or movement to a higher level of security. Meanwhile, lower scores on the different sets of SPJ guidance were associated with decreased likelihood of admission to secure services or lower security admission and movement to lower security levels. The DUNDRUM-1 was the most widely studied SPJ guidance for supporting admission decision-making, including selecting the most appropriate security level and movement between security levels. This is to be expected as the DUNDRUM-1 was specifically developed for this purpose. While the DUNDRUM-2 is intended to support admission decision-making alongside the DUNDRUM-1, specifically priority for admission, this was less widely investigated. In line with the wider pattern, DUNDRUM-1 and DUNDRUM-2 scores were predictive of decisions to admit overall, with higher scores indicating a greater need or priority for admission. The highest predictive accuracy of the DUNDRUM-1 for indicating admission need was observed in the initial validation study, based in the setting where the DUNDRUM was developed. This is the sole forensic mental health hospital in Ireland incorporating low, medium, and high secure services within the same setting [40, 48]. This represents a relatively unique context as, for example, in England and Wales until very recently the three high secure hospitals were separate to other security levels. The DUNDRUM-1 was predictive of secure service admission in England, Wales, and Belgium and able to distinguish between specific security levels. However, across these studies the DUNDRUM-1 did not consistently differentiate between admission to each security level. The ability to distinguish between security levels was most consistently observed for high security, followed by medium security, while low secure associations were less prevalent. This indicates that, in its current form, this guidance might not be directly applicable to all settings and systems. There is, however, scope for local developments that account for nuances and context of a particular setting.

The HCR-20 (V2 and V3), DUNDRUM-3 and DUNDRUM-4 were investigated for guiding service user movement decisions, with the HCR-20 Dynamic Scale (Clinical and Risk Scale combined), DUNDRUM-3 and DUNDRUM-4 found to be predictive of movement to higher or lower security levels. While sub-scales of the HCR-20 were to some extent relevant for movement to the most appropriate security level, the HCR-20 was not predictive across all sub-scales. One study explored whether the HCR-20 could guide initial admission decisions; the HCR-20 was not able to distinguish between those admitted or not admitted. When considering the findings, it is important to note the HCR-20 was not developed to inform admission decision-making and is more frequently applied to discharge decisions. While assessing the severity and imminence of risk potential is one consideration for deciding the most appropriate level of security there are also other factors unrelated to this that will influence this decision.

### Use of the SPJ approach for admission decision-making

There is an emerging evidence base about the use of the SPJ approach to support admission decision-making. However, the current evidence does not indicate the replacement of other decision-making processes with the use of SPJ guidance only. Across the evidence presented in this review, the admission recommendation based on the scoring of the studied SPJ guidance was compared to service user placement based on the usual decision-making process, such as court decision or clinical assessment. This demonstrates that SPJ guidelines can, at times, fit with existing decision-making, indicating potential value as a complementary approach. However, it does not provide evidence for the use of the SPJ approach in preference to existing procedures. While, theoretically, SPJ-informed guidance might be able to increase consistency and systematise decision-making, further investigations across a wider range of security levels, locations, and service types would be needed to confirm this. Establishing inter-rater and intra-rater reliability of SPJ guidance in the context of admission decision-making is also important. While inter-rater reliability was briefly examined for the DUNDRUM-1 [40, 49] and DUNDRUM-2 [48, 49] it was not widely explored across all SPJ guidelines or studies. This should include considerations of whether, as observed in one of the included studies, training in using a particular set of guidelines can improve reliability [49]. Developments in the consideration of protective factors, such as the Structured Assessment of Protective Factors for Violence Risk (SAPROF), could also be explored in the context of admission decision-making [54].

Contextual and service factors have a role in admission decision-making [15]. One such factor is location at referral, as highlighted within the included articles. Location at referral influenced whether scores on the chosen SPJ guidance (DUNDRUM-2, DUNDRUM-3, DUNDRUM-4 and HCR-20) could differentiate between service users, both for decisions regarding priority for admission and movement between security levels [46, 48]. Among high secure hospital referrals, those referred from prison were observed to have an increased need for admission, reflected through higher DUNDRUM-1 scores, than those referred from medium secure hospitals [45]. This raises the question of whether SPJ guidelines should take such factors into account, in line with the original purpose of the SPJ approach to account for context, or to improve equality regarding who is admitted, contextual factors should have a reduced role in admission decision-making. The interaction between such factors and SPJ guidelines during admission decision-making is an area requiring further exploration.

The evidence presented should be interpreted while considering the origins and subtle variations in what is meant by the SPJ approach. The integral element of the SPJ approach, as it was developed, is the use of clinical discretion while enabling consistency and systematisation [55]. These core principles form the basis of the HCR-20 and were used to develop the DUNDRUM toolkit. However, admission assessment research has focused solely on statistical associations with and predictive accuracy of service user placement or movement, as opposed to the integration of these guidelines into clinical practice. Throughout the research in this review, the chosen SPJ guidance was scored, with scores being compared and used to calculate predictive accuracy. While from a research perspective, this can provide a robust evidence base, it does not represent an SPJ-informed approach as initially intended, and in fact, more closely reflects an actuarial approach. This is a valuable first step in investigating the validity of these guidelines for admission assessments. However, the current evidence base does not reflect the context of intended use. To truly adhere to the principles of SPJ, future studies should examine application in clinical practice without focusing on scores and gain clinicians’ perspectives regarding the use and perceived acceptability of these guidelines.

### Limitations and future directions

Regarding limitations in the review methodology, a narrative synthesis addressed the aims of the review. However, if more research was available, across different settings and samples, a future systematic review using meta-analysis would be pertinent. A meta-analysis would enable the investigation of wider aims beyond the scope of individual studies, including how consistently SPJ guidelines are predictive of admission decision-making across a range of services and service user populations [56]. The robustness of the review is also, to some extent, affected by the limitations of the included papers. Not all study designs incorporated blinding to outcomes between those rating SPJ guidelines and making decisions regarding admission, this limits predictive validity as it is unclear whether lack of independent decision-making can explain agreement between the SPJ guidelines and alternative clinical assessment. Due to reporting errors and missing values required to interpret findings, some findings are omitted from the synthesis, to avoid false interpretation, but are highlighted in Table 3. Across the research, there were also differences in the scoring of the DUNDRUM-1, with mean score for each participant and total score for each participant both being used to calculate an overall average score. The authors of the DUNDRUM-1 recommend calculating a mean score for each service user to correspond with the most appropriate security level [25]. Seven studies did not score in this manner and deviation from intended scoring impacts the integrity of study findings. Inconsistencies in the total number of DUNDRUM-1 items used were also observed, with six studies using all 11 items whilst three used nine items, omitting two items relating to self-harm. By applying different parameters while using the same SPJ guidance, comparisons between findings are less robust as admission decisions are not based on the same DUNDRUM-1 criteria.

While both national and international research was identified, there was a limited number of articles. The identified research spanned five countries in total. There was one study in Canada, the remaining studies were conducted across Europe, this offers a Westernised, primarily Eurocentric perspective, limiting the international picture. With five studies in England and Wales, the national evidence is also based on a limited amount of research. Evaluating the use of SPJ-informed guidance for admission internationally offers information on application in different contexts, however, it is difficult to compare findings with a limited number of studies spread across different countries. The variability in service provision and decision-making further limits the extent of comparability. For example, in Belgium, only high secure services are obliged to accept all service users sent by the court, resulting in an increased likelihood of court recommendations for high security. As this policy does not apply to all countries included in the review, the decision-making processes may be context-dependent and influenced by different factors across studies. Multiple studies in a particular country could indicate how the application of SPJ guidelines may work nationally, and comparison of findings and national patterns across countries could then enhance the international evidence base. Specific researchers, in some instances those that developed the guidance, were consistently a part of much of the research in this review. Having experts within the research team, while to some extent helpful, limits understanding of how SPJ guidelines are used by researchers and, ultimately, clinicians that are less familiar with the guidance. The next step would be for more research teams to expand the investigation to new settings.

While the secure service population is predominantly male, reflected in the predominantly male sample included in much of the research, admission decisions for samples outside of the majority are equally important. One study included an all-female sample and small female samples were included in three studies. However, there is a clear gap in the research regarding the use of SPJ-informed guidance for admission among women. Secure service types include those specifically for women, with admission criteria differing from services for men, and it is unclear how applicable current SPJ guidelines would be for admission to women’s secure services. Women requiring secure service admission do not present in the same way as men, with an increased prevalence of borderline personality disorder, complex trauma, and disordered attachments, alongside the prevalence of serious self-injury or attempted suicide [17, 18]. While DUNDRUM-1 items related to self-harm were omitted from three studies due to previous findings of reduced association with placement, further research should explore whether these items may be more beneficial for women’s secure service admission assessments. There are a range of SPS for specific populations, including acquired brain injury, learning disability, autism, and personality disorder but there remains a gap regarding how SPJ guidelines could be used across these more specialist services. Insight from service users who have undergone admission assessments regarding their experiences would provide an important perspective and could guide developments in SPJ guidelines tailored to specific populations and their needs.

## Conclusions

While the SPJ approach is established in risk assessment and management, more recent applications to admission assessments have been less extensively explored. Across the included studies, SPJ guidance was able to match admission and movement decisions based on other approaches to decision-making. Overall, scores on SPJ guidance were positively associated with admission, security level selection and movement. However, the way the guidance was applied was not entirely reflective of SPJ core principles. Based on this evidence, it is not clear how best to use the SPJ approach for admission assessments and it cannot be concluded that SPJ use is preferable to other approaches for this purpose. Given the highlighted limitations, including the variability and sparsity of research, further evidence is needed before recommendations can be made. This would ideally expand on the current evidence base through increased research, both nationally and internationally, in different settings and with varied service user populations, reflecting how the SPJ approach would be used in these contexts. Gaining a clinician and service user perspective through qualitative research is also important to determine acceptability and whether those who would be using these guidelines feel they would be beneficial as part of their decision-making process.

## Supporting information

S1 Checklist Completed PRISMA checklist. (DOCX)

S1 Table MMAT quality appraisal for quantitative non-randomised research. (DOCX)

S2 Table MMAT quality appraisal for quantitative descriptive research. (DOCX)

S3 Table Item-level findings. (DOCX)

S1 Text Description of item-level findings. (DOCX)
