# Supplementary material for: Clinicians’ use of the structured professional judgement approach for adult secure psychiatric service admission assessments: A systematic review
Source: PLoS One. 2024 Sep 26;19(9):e0308598. doi: 10.1371/journal.pone.0308598 (PMC11426426; doi:10.1371/journal.pone.0308598)
Supplement: S1 Table — (DOCX) [file pone.0308598.s002.docx]

|  | | | | | | | |
| --- | --- | --- | --- | --- | --- | --- | --- |
| Supplementary Table 1. MMAT quality appraisal for quantitative non-randomised research | | | | | | | |
| Article  (Authors, year, title) | S1.  Are there clear research questions? | S2.  Do the collected data allow to address the research questions? | 3.1.  Are the participants representative of the target population? | 3.2.  Are measurements appropriate regarding both the outcome and intervention (or exposure)? | 3.3.  Are there complete outcome data? | 3.4.  Are the confounders accounted for in the design and analysis? | 3.5.  During the study period, is the intervention administered (or exposure occurred) as intended? |
| G. Flynn; C. O'Neill; C. McInerney; H. G. Kennedy. 2011. The DUNDRUM-1 structured professional judgment for triage to appropriate levels of therapeutic security: retrospective-cohort validation study. | Yes | Yes | Yes.  The target population was defined clearly as males from a specific prison that underwent psychiatric screening and required further assessment/support. The two samples consisted of those that were diverted from prison or identified for further psychiatric assessment during a specific time frame; however further sample characteristics were not reported. | Yes.  The two 3-point scales to measure service user outcomes were clearly stated, the DUNDRUM-1 was used in line with the research aims and the number of DUNDRUM-1 items used was stated. It should be noted that there are inconsistencies in the number of DUNDRUM-1 items used across studies. | Yes.  Retrospective design and complete outcome data for both samples*.* | Yes.  Those scoring the DUNDRUM-1 were blind to service user outcomes so could not be influenced by previous placement decisions and robust analysis methods were used. However, further considerations of confounds such as the influence of previous admission on decision-making could have been made. | Yes.  Participants were assessed retrospectively using the DUNDRUM-1 for comparison with placement outcomes. |
| D. Lawrence; T. L. Davies; R. Bagshaw; P. Hewlett; P. Taylor; A. Watt. 2018. External validity and anchoring heuristics: application of DUNDRUM-1 to secure service gatekeeping in South Wales. | Yes | Yes | Yes.  The sampling technique only included new referrals to psychiatric services during a specific time frame, establishing clear inclusion criteria; however, sample characteristics were not reported. | Yes.  The 5-point scale to measure service user outcomes was clearly stated and the DUNDRUM-1 was used in line with the research aims. It should be noted that there are inconsistencies in the number of DUNDRUM-1 items used across studies. | Yes.  Retrospective design and all those assessed appear to have been included. | Yes.  Only included first time referrals which removed potential impact of previous admission on decision-making, raters of the DUNDRUM-1 were not involved in referral and were blind to outcomes. | Yes.  Participants were assessed retrospectively using the DUNDRUM-1 for comparison with placement outcomes. |
| I. Jeandarme; P. Habets; H. Kennedy. 2019. Structured versus unstructured judgment: DUNDRUM-1 compared to court decisions. | Yes | Yes | Yes.  The sample was selected randomly from the target population of males referred by court to psychiatric services and descriptions of sample characteristics were included. | Yes.  The 5-point scale to measure service user outcomes was clearly stated and the DUNDRUM-1 was used in line with the research aims. Further information on how court decisions were made could be useful. It should be noted that there are inconsistencies in the number of DUNDRUM-1 items used across studies. | Yes.  There was data for 145/150 participants (5 participants missing due to incomplete DUNDRUM-1 or court data) which was 96.6% complete and judged to be satisfactory. | Cannot tell.  Those scoring the DUNDRUM-1 were blind to outcomes for those referred to various security levels and current placement for those referred to medium secure but not placement on admission. It is unclear based on the information if those scoring the DUNDRUM-1 were aware of exact admission decisions or more generally that referrals were to medium secure. Random sampling was used to control for confounds to some extent. | Yes.  Participants were assessed retrospectively using the DUNDRUM-1 for comparison with placement outcomes. |
| H. K. Williams; M. Senanayke; C. C. Ross; R. Bates; M. Davoren. 2020. Security needs among patients referred for high secure care in Broadmoor Hospital England | Yes | Yes | Yes.  Included all referrals (aside from one exclusion) to the target hospital for a specific time frame and provided information about the sample. | Cannot tell.  Define the DUNDRUM toolkit and state the number of DUNDRUM-1 items used but there was a lack of information regarding how service user outcomes were measured. It should be noted that there are inconsistencies in the number of DUNDRUM-1 items used across studies. | Yes.  All referrals assessed (aside from one exclusion) although the DUNDRUM-1 outcomes were reported more comprehensively than DUNDRUM-2. | Yes.  Blinding to outcomes across comparisons (DUNDRUM assessment, clinicians, and a panel) and rated independently. Although as there are only three high secure hospitals with specific criteria, service users may be referred/admitted multiple times and historic admissions could influence assessment. | Yes.  Participants were assessed retrospectively using the DUNDRUM-1 for comparison with standard admission outcomes. However, more information could be provided for the DUNDRUM-2 as only a pathway comparison was provided. |
| Habets, P., Jeandarme, I., & Kennedy, H. G. 2020. Determining security level in forensic psychiatry: a tug of war between the DUNDRUM toolkit and the HoNOS-Secure. | Yes | Yes | Yes.  Selected a random sample and checked that this sample was representative of the population being studied. | Yes.  The scales used to measure service user outcomes were clearly stated and the use of the DUNDRUM-1 and DUNDRUM-2 directly addresses the research aims. It should be noted that there are inconsistencies in the number of DUNDRUM-1 items used across studies. | Yes.  With the caveat that there was some missing data (still over 95% complete outcome data which was considered satisfactory) and it is not clear why. | Yes.  Utilised random sampling, blinding to outcomes, considered whether the sample was representative, chi squared tests used to check for confounds of age and CPS decision. | Yes.  It should be noted that one rater was not trained in using the DUNDRUM while the others were. |
| R. M. Jones; K. Patel; A. I. F. Simpson. 2019. Assessment of need for inpatient treatment for mental disorder among female prisoners: a cross-sectional study of provincially detained women in Ontario. | Yes | Yes | No.  Likert rating of sample selection limits how representative the sample can be by excluding those rated as having mild to no mental health needs and only including a small sample of those rated as having moderate mental health needs. | Yes.  The scales used to measure service user outcomes were clearly stated, DUNDURUM-1 and DUNDRUM-2 were appropriate measures in line with the research aims. It should be noted that there are inconsistencies in the number of DUNDRUM-1 items used across studies. | Yes.  Of those that were included, there was complete outcome data with three missing files (remains over 95% complete outcome data which was judged to be satisfactory). | No.  The design had potential for confounds due to the sample selection (selecting participants on a likert scale based on 'severity') and it was unclear if the DUNDRUM was rated independently and blind to outcomes. The moderate cases and missing data were prorated. However, it was assumed that no cases assessed as having ‘mild’ or ‘none’ mental health needs would require admission. | Yes.  The DUNDRUM-1 and DUNDRUM-2 were rated and compared to a clinical assessment. |
| Flynn G, O’Neill C, Kennedy HG. 2011. DUNDRUM-2: Prospective validation of a structured professional judgment instrument assessing priority for admission from the waiting list for a forensic mental health hospital. | Yes | Yes | Yes.  The target population was those on the waiting list for admission and included all service users on the waiting list during a specific time frame. Should be noted that the sample sizes do not add up and a more detailed description of the sample would be beneficial. | Cannot tell.  The scale used to measure service user outcomes were clearly stated for the DUNDRUM-2 but not the DUNDRUM-1. The DUNDRUM-1 and DUNDRUM-2 were measured in line with the research aims. It should be noted that there are inconsistencies in the number of DUNDRUM-1 items used across studies. | Yes.  Outcome data included for all those on the waiting list during the 6-month period. | Yes.  Those making admissions decisions were blind to existing DUNDRUM ratings and 24 cases were rated independently by a second clinician. Conducted comparisons across locations to determine if those from a particular setting are more likely to be admitted. | Yes.  The DUNDRUM-1 and DUNDRUM-2 was rated weekly and compared to decisions to admit. |
| M. Freestone; D. Bull; R. Brown; N. Boast; F. Blazey; P. Gilluley. 2015. Triage, decision-making and follow-up of patients referred to a UK forensic service: validation of the DUNDRUM toolkit. | Yes | Yes | Yes.  Representative of a population referred to low/medium secure services and to some extent high secure (referrals to and from high secure were excluded from AUC analysis) during a specific time frame, include sample characteristics and a diagram detailing how many participants completed each stage alongside any reasons for exclusion. | Cannot tell.  The scales used to measure service user outcomes were not clearly defined. Measured the DUNDRUM-1 and DUNDRUM-2 with comparisons to clinical assessment which was appropriate for the aims. It should also be noted that there are inconsistencies in the number of DUNDRUM-1 items used across studies. | Yes.  No loss of outcome data for those included for admission assessment, some loss to follow-up however this is not within the scope of the review. | No.  The researchers highlight potential limitations in the design including the lack of blinding resulting in potential for confirmation bias and a halo effect. This limits how much can be assumed about predictive validity from this study as due to the lack of independence between the variables being compared one may have affected the other explaining agreement between the SPJ guidelines and current practice. | Yes.  The DUNDRUM-1 and DUNDRUM-2 were rated and compared to clinical decisions (although with the caveat this was not independent). |
| Davoren, M., O'Dwyer, S., Abidin, Z., Naughton, L., Gibbons, O., Doyle, E., McDonnell, K., Monk, S., & Kennedy, H. G. 2012. Prospective in-patient cohort study of moves between levels of therapeutic security: The DUNDRUM-1 triage security, DUNDRUM-3 programme completion and DUNDRUM-4 recovery scales and the HCR-20. | Yes | Yes | Yes.  Included inpatients at the central mental hospital during a specific time frame and described sample characteristics. | Cannot tell.  Clearly defined outcome measures and scoring for the DUNDRUM-1 however scoring of outcomes for other measures was not provided. The use of the DUNDRUM-1 DUNDRUM-3, DUNDRUM-4, and HCR-20 was justified based on research aims. | Yes.  Appears that data for all eligible participants was included throughout the study. | Yes.  Consider confounds and include extra measures to account for potential confounds, both in design and in the analysis with analyses including location at baseline as a covariate. | Yes.  Rated patients on the measures based on clinical audit data and then observed movement between security levels. |
| Davoren, M., Hennessy, S., Conway, C., Marrinan, S., Gill, P., & Kennedy, H. G. 2015. Recovery and concordance in a secure forensic psychiatry hospital–the self rated DUNDRUM-3 programme completion and DUNDRUM-4 recovery scales. | Yes | Yes | Yes.  Included inpatients at the target hospital during a specific time frame and described sample characteristics. | Cannot tell.  Stated outcome as inpatient movement but did not provide details regarding how outcome was measured. The DUNDRUM-3, DUNDRUM-4, and HCR-20 use was justified based on research aims. | Yes.  For the clinician-rated measures there was a full sample and no missing data, which is the data extracted for the purposes of this review. Not all components of the study had complete data, but this was beyond the scope of this review. | Yes.  Clinicians scoring the guidelines were blind to outcomes and those making decisions were not aware of scores. Analysis included regression but did not include covariates such as location at baseline. The authors themselves highlight potential confounds including selective placement over time. | Yes.  Clinicians rated the measures and then movement between security levels was observed. |
| McCullough, S., Stanley, C., Smith, H., Scott, M., Karia, M., Ndubuisi, B., ... & Davoren, M. 2020. Outcome measures of risk and recovery in Broadmoor High Secure Forensic Hospital: stratification of care pathways and moves to medium secure hospitals. | Yes | Yes | Yes.  Include the whole sample from the target hospital during a specific time frame. | Cannot tell.  Stated outcome as inpatient movement to lower security but did not provide details regarding how outcome was measured. Use of the DUNDRUM-3, DUNDRUM-4 and HCR-20 was in line with the aims and justified. | Yes.  Data was complete for all service users involved in the study. | No.  While confounds are acknowledged retrospectively in the discussion, they are not controlled for in design/analysis which is what this criterion addresses. | Cannot tell.  There is a lack of detail, specifically whether all service users’ follow up lasted 13 months as the study start is listed as May and June 2016 and ended in June 2017. |
