# Supplementary material for: Clinicians’ use of the structured professional judgement approach for adult secure psychiatric service admission assessments: A systematic review
Source: PLoS One. 2024 Sep 26;19(9):e0308598. doi: 10.1371/journal.pone.0308598 (PMC11426426; doi:10.1371/journal.pone.0308598)
Supplement: S2 Table — (DOCX) [file pone.0308598.s003.docx]

| Supplementary Table 2. MMAT quality appraisal for quantitative descriptive research | | | | | | | |
| --- | --- | --- | --- | --- | --- | --- | --- |
| Article  (Authors, year, title) | S1.  Are there clear research questions? | S2.  Do the collected data allow to address the research questions? | 4.1.  Is the sampling strategy relevant to address the research question? | 4.2.  Is the sample representative of the target population? | 4.3.  Are the measurements appropriate? | 4.4.  Is the risk of nonresponse bias low? | 4.5.  Is the statistical analysis appropriate to answer the research question? |
| C. Duggan; L. Mason; P. Banerjee; J. Milton. 2007. Value of standard personality assessments in informing clinical decision - making in a medium secure unit. | Yes | Yes | Cannot tell.  Exclusions are not explained clearly in the paper and seems other sampling strategies may have been more suitable but then this is potentially just following service criteria. | No.  While the focus was on a specific service sample relevant to the target population and sample characteristics were reported with some justification of exclusions, there are four service users missing from the sample that there is no reason for exclusion. Listed reasons for exclusion also include personality disorder which is confusing due to this being the target population. | Yes.  Use validated/ standardised measures with admission as the outcome. | Not applicable. | Cannot tell.  Very limited analyses presented. |
