# Supplementary material for: Clinicians’ use of the structured professional judgement approach for adult secure psychiatric service admission assessments: A systematic review
Source: PLoS One. 2024 Sep 26;19(9):e0308598. doi: 10.1371/journal.pone.0308598 (PMC11426426; doi:10.1371/journal.pone.0308598)
Supplement: S3 Table — (DOCX) [file pone.0308598.s004.docx]

| Supplementary table 3. Item-level findings | | | | |
| --- | --- | --- | --- | --- |
| Author(s) | Year | Study title | Aims and outcome measures related to item-level findings | Item-level findings |
| G. Flynn; C. O'Neill; C. McInerney; H. G. Kennedy | 2011 | The DUNDRUM-1 structured professional judgment for triage to appropriate levels of therapeutic security: retrospective-cohort validation study | To examine the relationship between individual items of the DUNDRUM-1 and patient placement  Item based outcomes measured on a 4-point scale:  (0) Not admitted.  (1) Admitted to an open ward.  (2) Admitted to a PICU  (3) Admitted to the Central Mental Hospital (medium/high) | DUNDRUM-1 item correlation with patient placement for the total sample:  Item 1 (Serious violence; rs=0.80, p<0.001),  Item 2 (Serious self-harm; rs=0.26, p<0.001)  Item 3 (Immediacy of violence risk; rs=0.88, p<0.001),  Item 4 (Immediacy of self-harm risk; rs=0.24, p<0.001),  Item 5 (Specialist forensic need; rs=0.91, p<0.001),  Item 6 (Absconding risk; rs=0.88, p<0.001),  Item 7 (Preventing access; rs=0.83, p<0.001),  Item 8 (Victim sensitivities; rs=0.81, p<0.001),  Item 9 (Complex risks; rs=0.83, p<0.001),  Item 10 (Institutional behaviour; rs=0.76, p<0.001),  Item 11 (Legal procedure; rs=0.92, p<0.001) |
| D. Lawrence; T. L. Davies; R. Bagshaw; P. Hewlett; P. Taylor; A. Watt | 2018 | External validity and anchoring heuristics: application of DUNDRUM-1 to secure service gatekeeping in South Wales | To examine the association between individual items of the DUNDRUM-1 and patient placement.  ROC and whether the AUC indicated that individual DUNDRUM-1 items distinguished between those admitted to low/medium secure and those not admitted. | DUNDRUM-1 items and security level required:  Analysis of each item observed that patients placed at higher security levels had significantly higher scores across five items including:  Item 1 (Seriousness of violence; p<.000), Item 3 (Immediacy of risk of violence; p<.000), Item 5 (Specialist forensic need; p<.000), Item 11 (Legal process; p<.000) and Item 10 (Institutional behaviour; p<.001)  DUNDRUM-1 items predictive validity:  ROC:  There were five items predictive of admission to secure services (low/medium secure):  Immediacy of risk of violence (item 3): AUC 0.72 (95%CI 0.62-0.83)  Specialist forensic need (item 5): AUC 0.71 (95%CI 0.61-0.82)  Absconding (item 6): AUC 0.65 (95%CI 0.53-0.78)  Institutional behaviour (item 10): AUC 0.67 (95%CI 0.54-0.79)  Legal process (item 11): AUC 0.90 (95%CI 0.72-0.98) ^d^  The remaining six items had lower bounds of 95% confidence intervals overlapping/falling below 0.5, indicating predictive validity no better than chance. |
| I. Jeandarme; P. Habets; H. Kennedy | 2019 | Structured versus unstructured judgment: DUNDRUM-1 compared to court decisions | Comparison of court determined patient placement and scores on individual DUNDRUM-1 items. ROC and whether the AUC indicated that individual DUNDRUM-1 items distinguished between different security levels. | DUNDRUM-1 items and required security level:  Comparison of mean scores on each DUNDRUM-1 item per court decision indicated that there was a significant difference between groups overall for:  Item 1 (Seriousness of violence), Item 3 (Immediacy of risk of violence), Item 5 (Specialist forensic need) Item 6 (Absconding/eloping) and Item 8 (Victim sensitivity/public confidence issues) and Item 11 (Legal process), Kruskal Wallis, p < 0.05).  Post-hoc comparisons indicated significant differences between groups admitted to different security levels for two items:  Item 6 (Absconding/eloping):  Open v. high (p<0.01)  Medium v. high (p<0.01).  Item 11 (Legal process):  Ambulatory and medium (p<0.01),  Ambulatory and high (p<0.01)  Ambulatory and open (p<0.01)  Medium and high (p<0.01). ^b^  (Mean scores were not reported to indicate which admission settings had the higher scores).  DUNDRUM-1 items predictive validity  ROC:  Specialist forensic need (AUC=0.64; p < 0.05)  Absconding/eloping (AUC=0.71; p < 0.05)  Legal process (AUC=0.81; p < 0.05). ^b^ |
| P. Habets, I. Jeandarme, & H.G. Kennedy. | 2020 | Determining security level in forensic psychiatry: a tug of war between the DUNDRUM toolkit and the HoNOS-Secure. | ROC and whether the AUC indicated that individual items on the DUNDRUM-1 distinguished between different security levels. | DUNDRUM-1 items predictive validity  ROC:  Two items were predictive of high-secure court decision:  Absconding/eloping (AUC = 0.62; p < 0.02)  Legal process (AUC = 0.74; p < 0.01). ^b^ |
| R. M. Jones; K. Patel; A. I. F. Simpson | 2019 | Assessment of need for inpatient treatment for mental disorder among female prisoners: a cross-sectional study of provincially detained women in Ontario | The relationship between likelihood of admission and scores on individual DUNDRUM-1 and DUNDRUM-2 items. | DUNDRUM-1 items:  Need for admission was significantly related to scores on six DUNDRUM-1 items, with likelihood of admission increasing as scores increased across Item 1 (Seriousness of violence), OR= 2.07 (95%CI 1.30-3.30), p=0.002, Item 3 (Immediacy of violence risk), OR= 2.68 (95%CI 1.62- 4.43), p<0.001, Item 5 (Specialist forensic need), OR= 5.04 (95%CI 1.83-11.45), p<0.001, Item 6 (Absconding/eloping), OR= 1.95 (95%CI 1.15-3.30), p=0.013, Item 9 (Complex needs re violence), OR= 2.43 (95%CI 1.44-4.12), p=0.001 and Item 10 (Institutional Behaviour), OR= 4.49 (95%CI 2.17-9.27), p<0.001.^c^  DUNDRUM-2 items:  Need for admission was also significantly related to scores on four DUNDRUM-2 items, with likelihood of admission increasing as scores increased across Item 1 (Urgency: Remand/sentenced prisoner), OR= 3.02 (95%CI 1.77-5.14), p<0.001, Item 2 (Mental health), OR= 7.59 (95%CI 2.96-19.45), p<0.001, Item 4 (Humanitarian), OR= 4.01 (95%CI 2.17-7.40), p<0.001 and Item 5 (Systemic), OR= 3.73 (95%CI 2.12-6.73), p<0.001. ^c^ |
| M. Freestone; D. Bull; R. Brown; N. Boast; F. Blazey; P. Gilluley | 2015 | Triage, decision-making and follow-up of patients referred to a UK forensic service: validation of the DUNDRUM toolkit | ROC and whether the AUC indicated that individual DUNDRUM-1 items distinguished between those admitted to low or medium secure services and those not admitted. | DUNDRUM-1 items predictive validity:  Items that were predictive of admission to forensic low secure or medium secure were:  Seriousness of violence (item 1; AUC= 0.70, 95%CI 0.62-0.77)  Immediacy of violence (item 3; AUC=0.76 95%CI 0.69-0.83)  Specialist forensic need (item 5; AUC= 0.72, 95%CI 0.64-0.79)  Absconding/eloping (item 6; AUC=0.69, 95%CI 0.61-0.76)  Preventing access (item 7; AUC= 0.69, 95%CI 0.61-0.76)  Victim sensitivity (item 8; AUC= 0.64, 95%CI 0.56-0.72)  Complex risk of violence (item 9; AUC= 0.62, 95%CI 0.54-0.70)  Legal process (item 11; AUC= 0.76, 95%CI 0.69-0.83). |
| G, Flynn., C, O’Neill., H.G, Kennedy | 2011 | DUNDRUM-2: Prospective validation of a structured professional judgment instrument assessing priority for admission from the waiting list for a forensic mental health hospital | Comparison of scores on individual DUNDRUM-2 items for those prioritised for admission or not admitted.  Comparison of DUNDRUM-1 scores for those admitted and not admitted. | DUNDRUM-2 items and priority for admission:  Patients admitted had significantly higher scores than those not admitted across four items:  Item 1 (Location), F=49.3, (df=1), p<0.001  Item 2 (Mental health), F=23.1, (df=1), p<0.001  Item 4 (Humanitarian), F= 28.6, (df=1), p<0.001  Item 5 (Systemic), F= 31.3, (df=1), p<0.001  Higher scores were significantly associated with admission across four items, Item 1 (Location, X^2^ = 41.3, df=4, p<0.001), Item 2 (mental health, X^2^ = 20.0, df=4, p<0.001), Item 4 (Humanitarian, X^2^ = 20.9, df=4, p<0.001) and Item 5 (Systemic, X^2^ = 25.6, df=4, p<0.001).  Two items (Item 3, Self-harm and Item 6, Legal urgency) did not meet significance at a cut-off of p < 0.05.  DUNDRUM-1 items and need for admission:  Patients that were admitted had significantly higher scores than those not admitted on nine items:  Item 1 (Seriousness of violence), F= 13.2 (df=1), p<0.001, Item 3 (Immediacy of violence), F= 14.9 (df=1), p<0.001, Item 5 (Special forensic need), F= 15.9, (df=1), p<0.001, Item 6 (Absconding), F=6.8, (df=1), p<0.05, Item 7 (Preventing access), F=7.3, (df=1), p<0.01, Item 8 (Victim issues), F=7.8, (df=1), p<0.01, Item 9 (Risk of violence), F=12.2, (df=1), p<0.001, Item 10 (Institutional behaviour), F=3.9 (df=1), p<0.05, Item 11 (Legal procedure), F=43.9, (df=1), p<0.001  Higher scores on seven items were significantly associated with admission: Item 1 (Seriousness of violence; X^2^ = 14.7, df=4, p<0.01), Item 3 (Immediacy of violence; X^2^ = 13.6, df=4, p<0.01 ), Item 5 (Special forensic need; X^2^ = 13.5, df=4, p<0.01), Item 6 (Absconding; X^2^ = 10.1, df=4, p<0.05), Item 8 (Victim issues; X^2^ = 14.0, df=4, p<0.01), Item 9 (Risk of violence; X^2^ = 11.5, df=4, p<0.05), with Item 11 (Legal procedure) having the strongest association (X^2^= 32.4, df=4, p<0.001). |

*Please note. Throughout the table only item-level findings relevant to the review research question were included in the table.*

*Note ^a.^* All figures are reported to 2 decimal places (where provided in the original article) aside from p values reported up to 3 decimal places.

*Note ^b^*. All statistics not reported in the paper.

*Note ^c.^* The statistics presented in the table did not match those reported in the text, the statistics reported in this review are those presented in the table.

*Note ^d^.* Values (including means, AUCs and 95% CIs) are approximate due to extraction from a figure.

*Note ^e^*. The sample sizes do not add up.
