# Supplementary material for: Clinicians’ use of the structured professional judgement approach for adult secure psychiatric service admission assessments: A systematic review
Source: PLoS One. 2024 Sep 26;19(9):e0308598. doi: 10.1371/journal.pone.0308598 (PMC11426426; doi:10.1371/journal.pone.0308598)
Supplement: S1 Text — (DOCX) [file pone.0308598.s005.docx]

**Supplementary information: Item-level findings**

**DUNDRUM-1 items**

***Need for admission***

Two studies examined need for admission on an item level for the DUNDRUM-1 [43, 48]. One study observed that those admitted to secure psychiatric services had significantly higher scores on nine DUNDRUM-1 items compared to those not admitted: Item 1, (Seriousness of violence; F= 13.2 (df=1), p<0.001); Item 3 (Immediacy of violence; F= 14.9 (df=1), p<0.001); Item 5 (Special forensic need; F= 15.9, (df=1), p<0.001); Item 6 (Absconding; F=6.8, (df=1), p<0.05); Item 7 (Preventing access; F=7.3, (df=1), p<0.01); Item 8 (Victim issues ; F=7.8, (df=1), p<0.01); Item 9 (Risk of violence ; F=12.2, (df=1), p<0.001); Item 10 (Institutional behaviour ; F=3.9 (df=1), p<0.05); Item 11 (Legal procedure; F=43.9, (df=1), p<0.001) [48].

This study also observed that higher scores on seven items were significantly associated with admission from the waiting list: Item 1 (Seriousness of violence ; X^2^ = 14.7, df=4, p<0.01); Item 3 (Immediacy of violence; X^2^ = 13.6, df=4, p<0.01 ); Item 5 (Special forensic need; X^2^ = 13.5, df=4, p<0.01); Item 6 (Absconding; X^2^ = 10.1, df=4, p<0.05); Item 8 (Victim issues; X^2^ = 14.0, df=4, p<0.01); Item 9 (Risk of violence; X^2^ = 11.5, df=4, p<0.05); Item 11 (Legal procedure) showed the strongest association between score and admission (X^2^= 32.4, df=4, p<0.001) [48]. Likewise another study observed that likelihood of admission increased as scores on DUNDRUM-1 items increased across five of the same items (Items 1, 3, 5, 6, 9) and one additional item (Item 10): Item 1 (Seriousness of violence; OR= 2.07 95%CI 1.30-3.30, p=0.002); Item 3 (Immediacy of violence risk; OR= 2.68 95%CI 1.62- 4.43, p<0.001);Item 5 (Specialist forensic need; OR= 5.04 95%CI 1.83-11.45, p<0.001);Item 6 (Absconding/eloping; OR= 1.95 95%CI 1.15-3.30, p=0.013); Item 9 (Complex needs re violence; OR= 2.43 95%CI 1.44-4.12, p=0.001) and Item 10 (Institutional Behaviour; OR= 4.49 95%CI 2.17-9.27, p<0.001) [43].

***Required security level***

Three studies examined whether DUNDRUM-1 item scores could differentiate between those requiring a variety of security levels [40; 42; 44]. One study observed that the security level service users were placed in was significantly related to scores across all DUNDRUM-1 items, with those placed at a higher security level scoring significantly higher on DUNDRUM-1 items, the strongest associations was observed for items: Item 11 (Legal procedure; r_s_=0.92, p<0.001) ; Item 5 (Specialist forensic need; r_s_=0.91, p<0.001); Item 3 (Immediacy of violence risk; r_s_=0.88, p<0.001); Item 6 (Absconding risk; r_s_=0.88, p<0.001); Item 7 (Preventing access; r_s_=0.83, p<0.001); Item 9 (Complex risks; r_s_=0.83, p<0.001); Item 8 (Victim sensitivities; r_s_=0.81, p<0.001); Item 1 (Serious violence; r_s_=0.80, p<0.001) and Item 10 (Institutional behaviour; r_s_=0.76, p<0.001) [40]. A weak correlation was observed for Item 2 (Serious self-harm; r_s_=0.26, p<0.001) and Item 4 (Immediacy of self-harm risk; r_s_=0.24, p<0.001). Two further studies examined how service users placed across security levels may differ in DUNDRUM-1 score on an item level [42,44]. One study observed that service users placed at higher security levels had significantly higher DUNDRUM-1 item scores across five items including: Item 1 (Seriousness of violence; p<.000), Item 3 (Immediacy of risk of violence; p<.000), Item 5 (Specialist forensic need; p<.000), Item 11 (Legal process; p<.000) and Item 10 (Institutional behaviour; p<.001) [44]. The second study, comparing DUNDRUM-1 item scores to court decisions, observed a significant difference in scores across court decisions overall for four of the same items, including Item 1 (Seriousness of violence), Item 3 (Immediacy of risk of violence), Item 5 (Specialist forensic need) and Item 11 (Legal process), alongside Item 6 (Absconding/eloping) and Item 8 (Victim sensitivity/public confidence issues) (Kruskal Wallis, p < 0.05) [42]. The ability to differentiate between service user placement to specific security levels was observed for two items, including Item 6 (Absconding/eloping) when comparing open and high security (p<0.01) or medium and high security (p<0.01) and Item 11 (Legal process) when comparing ambulatory and medium (p<0.01), ambulatory and high (p<0.01) or medium and high security (p<0.01), although the direction of these differences was also not indicated [42].

**Predictive validity**

Four studies explored the predictive validity of individual DUNDRUM-1 items (41, 42, 44, 49). Two items (Item 6, Absconding/eloping and Item 11, Legal process) were predictive of admission across the four studies including admission to low/medium secure services combined, item 6 AUC = 0.65 (95% CI 0.52 - 0.78) - AUC=0.69 (95% CI 0.61 - 0.76); item 11 AUC= 0.76 (95% CI 0.69 -0.83)- AUC= 0.90 (95% CI 0.72 - 0.98) [41, 44] and high secure services, Item 6 AUC= 0.62, p<0.002 - AUC=0.71, p<0.05; item 11 AUC = 0.74 p<0.01- AUC=0.81 p<0.05 [42, 49].

**DUNDRUM-2 items**

**Priority for admission**

One study examined whether priority for admission was associated with DUNDRUM-2 scores on an item level (48). Those admitted scored as higher priority, with significantly higher scores than those not admitted (p<0.001) on all items except item 3 (Self-harm) and item 6 (Legal urgency) [48]. Admission from the waiting list was significantly associated with higher scores indicating higher priority on four items: Item 1 (Location, X^2^ = 41.3, df=4, p<0.001); Item 2 (Mental health, X^2^ = 20.0, df=4, p<0.001); Item 4 (Humanitarian, X^2^ = 20.9, df=4, p<0.001) and Item 5 (Systemic, X^2^ = 25.6, df=4, p<0.001) [48].

**Need for admission**

One study, which measured need for admission as the outcome for the DUNDRUM-2, observed that need for admission was significantly related to four items. Higher scores indicated increased likelihood of admission for Item 1 (Urgency: Remand/sentenced prisoner; OR 3.02, 95%CI 1.77-5.14 p<0.001), Item 2 (Mental health; OR 7.59 95%CI 2.96-19.45, p<0.001), Item 4 (Humanitarian; OR 4.01 95%CI 2.17-7.40, p<0.001) and Item 5 (Systemic, OR 3.73 95%CI 2.12-6.73, p<0.001 [43].
